# Nanozyme as detector and remediator to environmental pollutants: between current situation and future prospective

**DOI:** 10.1007/s11356-023-31429-0

**Published:** 2023-12-23

**Authors:** Hager A. Elkomy, Shimaa A. El-Naggar, Mariam A. Elantary, Sherif M. Gamea, Mahmoud A. Ragab, Omar M. Basyouni, Moustafa S. Mouhamed, Fares F. Elnajjar

**Affiliations:** 1 Biochemistry Sector, Chemistry Department, Faculty of Science, Tanta University, Tanta, 31527 Egypt; 2 Chemistry/Biochemistry Sector, Chemistry Department, Faculty of Science, Tanta University, Tanta, 31527 Egypt; 3 Chemistry/Zoology Sector, Chemistry Department, Faculty of Science, Tanta University, Tanta, 31527 Egypt; 4 Microbiology Sector, Faculty of Science, Tanta University, Tanta, 31527 Egypt

**Keywords:** Nanozyme, Environmental monitoring, Toxic ion pesticide, Pathogen, Antibiotic residue, Phenolic pollutant, Colorimetric detection, Fluorescent analysis

## Abstract

The term “nanozyme” refers to a nanomaterial possessing enzymatic capabilities, and in recent years, the field of nanozymes has experienced rapid advancement. Nanozymes offer distinct advantages over natural enzymes, including ease of production, cost-effectiveness, prolonged storage capabilities, and exceptional environmental stability. In this review, we provide a concise overview of various common applications of nanozymes, encompassing the detection and removal of pollutants such as pathogens, toxic ions, pesticides, phenols, organic contaminants, air pollution, and antibiotic residues. Furthermore, our focus is directed towards the potential challenges and future developments within the realm of nanozymes. The burgeoning applications of nanozymes in bioscience and technology have kindled significant interest in research in this domain, and it is anticipated that nanozymes will soon become a topic of explosive discussion.

**Graphical Abstract:**

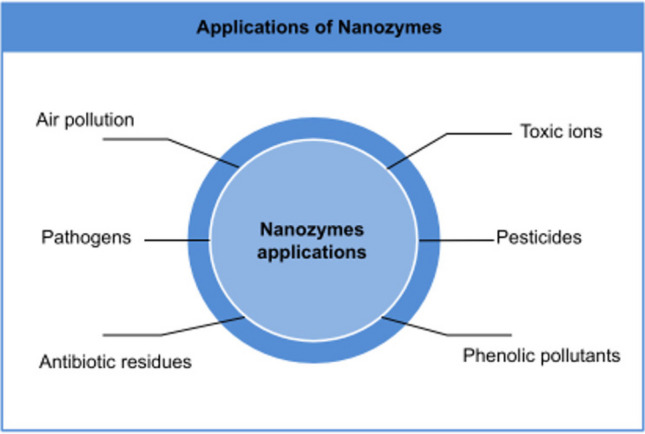

## Introduction

The inherent properties of natural enzymes endow them with remarkable biocatalytic efficiency, resulting in a wide range of significant applications in sensing, biomedicine, chemical synthesis, ecological engineering, food production, and processing. This efficacy arises from their exceptional substrate specificity and their ability to catalyze biological processes with high efficiency (Cramer & Kampe 1965; Orgel 1968). Despite encountering challenges such as instability, reduced catalytic efficiency under extreme conditions, complex preparation, expensive production, and difficulties in purification and recovery, natural enzymes have found extensive use. However, considering these limitations, artificial enzymes have emerged as promising alternatives. Over the past few decades, substantial progress has been achieved in the development of novel and efficient artificial enzymes, leading to remarkable advancements and notable accomplishments (Breslow 1995; Dong et al. 2012; Murakami et al. 1996). The fusion of biology and nanotechnology has resulted in significant strides in the creation of artificial enzymes, commonly known as nanozymes, composed of functional nanomaterials. These nanozymes offer significant advantages over natural enzymes and other artificial counterparts, including enhanced environmental stability, appropriate catalytic activity, and straightforward synthesis methods. Additionally, nanozymes can be produced on a larger scale and at a lower cost. Intriguingly, the unique physicochemical properties of nanoscale materials provide nanozymes with a diverse array of opportunities for functionalization and modification (X. Li et al. 2019a, b, c; Song et al. 2019; Wang et al. 2018). Commonly encountered nanozymes include layered double hydroxides (LDHs), metal oxides, metal chalcogenides, metal–organic frameworks (MOFs), metals, and nanocarbon materials (Song et al. 2019). Nanozymes have demonstrated a multitude of prospective applications across various research domains, encompassing sensing, antioxidant systems, immunoassays, disease diagnostics, defense mechanisms, and pollutant removal. The integration of nanozymes holds the potential to significantly enhance the efficacy of future environmental monitoring endeavors.

In the context of environmental applications, the systematic review of nanozymes has unveiled their pivotal role in addressing environmental challenges. Nanozymes have shown remarkable promise in sensing and eliminating environmental pollutants, including pathogens, toxic ions, pesticides, phenolic pollutants, and antibiotic residues. Their unique catalytic properties and stability under various environmental conditions make them ideal candidates for environmental remediation.

This comprehensive review delves into the principles and methodologies explored for the detection and remediation of prevalent pollutants, encompassing pathogens, toxic ions, pesticides, phenolic pollutants, and antibiotic residues. Furthermore, the review encompasses future prospects and emerging trends concerning the design and utilization of nanozymes within this field. Finally, the article extensively addresses the forthcoming challenges and perspectives associated with nanozymes, offering a comprehensive conclusion.

## Organic pollutants

Organic pollutants pose significant hazards due to their ability to accumulate and persist in various plant and animal ecosystems, including within organisms themselves. They give rise to a multitude of environmental issues and are associated with a range of serious diseases. These health problems encompass diabetes, cancer, cardiovascular disorders, endocrine disruptions, obesity, and reproductive issues (Alharbi et al. 2018). The removal of these contaminants can be achieved by employing metal compounds in the form of nanozymes. Utilizing metallic compounds as nanozymes facilitates the removal of organic pollutants effectively from the environment (Bethi et al. 2016). Fe_3_O_4_ nanoparticles exhibit catalytic magnetic activity akin to the enzyme peroxidase. They possess the ability to activate hydrogen peroxide and generate hydroxyl radicals, thus enabling the oxidation of substrates (Gao et al. 2007).

### *CoFe*_*2*_*O*_*4*_* nanozyme*

The paramount characteristic of this compound lies in its cost-effectiveness, efficiency, and exceptional stability. Its utility extends to the detection of hydrogen peroxide (H_2_O_2_) and the degradation of methylene blue (MB) dye due to its peroxidase-like activity (Wu et al. 2018). The key advantage of utilizing these nanoparticles lies in their porous nature, which significantly enhances their efficiency in the removal of organic pollutants from the environment (Wu et al. 2018).

### *Fe*_*3*_*O*_*4*_*@gel nanozyme*

Enhancing their effectiveness as nanoparticles, they can be coated with polyacrylamide. This coating enables them to eliminate approximately 99% of organic pigments, such as carmine (IC) and indigo, using oxidizing agents like H_2_O_2_ or Na_2_S_2_O_8_. The nanogel polymer absorbs the dyes effectively. The nanogel, composed of polyacrylamide with numerous amide groups, forms hydrogen bonds with IC, which contains two sulfonic groups. As the concentration of IC increases, the oxidation performance improves, and it is advisable to employ Fe_3_O_4_/Na_2_S_2_O_8_ due to its greater strength, attributed to the extended half-life of sulfate radicals, which enhances their impact on the dyes (Zha et al. 2022).

There are also many nano particles that have activity similar to the enzyme peroxidase such as, such as that CeO_2_ (Li et al. 2018), CuFe_2_O_4_ (Y. Wei et al. 2019a, b), FeS (Fan et al. 2018), CdS (Shen et al. 2015), MnO_2_ (Pan et al. 2021), and CuO-Fe_3_O_4_ have demonstrated a similar peroxidase like activity as Fe_3_O_4_ (Lei et al. 2015). And once oxidized and in the presence of persulfate, peroxymonosulfate, or hydrogen peroxide, these substances can effectively remove organic pollutants (He et al. 2021; Zhou et al. 2018).

### FeBi-NC nanozyme

This material is synthesized from Fe-doped Bi-MOF. The presence of BiN_4_ and Fe-N_4_ sites is confirmed through EXAFS and XANES analysis. Notably, it exhibits high single atom loadings of Bi (8.01 wt%) and Fe (2.61 wt%). Moreover, it demonstrates catalytic activity comparable to that of the oxidase (OXD) enzyme (Chen et al. 2022). This material finds application in the elimination of organic pollutants and displays impressive efficacy. It achieves complete removal (100%) of rhodamine B dye, removes 96% of methyl orange pigment within 1 min, and eliminates 95% of methylene blue in just 2 min. These results underscore its remarkable capability for pollutant removal (Chen et al. 2022).

### Au-NiFe layered double hydroxide LDH/rGO

The Au-NiFe LDH/rGO nanoparticle represents a novel material proficient in the efficient decomposition of organic mercury. Its mechanism involves the creation of an amalgam on the gold atoms’ surface, thereby expediting electron transfer and the generation of CH_3_ radicals from the methyl group in MeHg, as well as O^−2^ radicals from oxygen. This catalytic process closely resembles the activity of the oxidase (OXD) enzyme. Remarkably, this nanoparticle can analyze and eliminate 99.9% of organic mercury within a mere two-hour timeframe, all without the release of any harmful mercury ions (H. Liu et al. 2021c, d, e, f, a, b).

### *Fe*_*3*_*O*_*4*_* nanoparticles decorated functionalized rGO sheets nanozymes*

FDG nanozymes exhibit dual functionality in both detecting and assessing the adverse impacts of the simazine pesticide. Their operation relies on the establishment of hydrogen bonds between TMB (3,3′,5,5′ tetramethyl benzidine) and pesticide molecules, which hampers the nanozyme’s activity and halts the oxidation of TMB. Consequently, the presence of the pesticide in the water medium can be readily ascertained. Furthermore, simazine can undergo photocatalytic degradation, and notably, exceptional degradation efficiency is achieved under direct sunlight irradiation. Additionally, FDGs can be reused multiple times in the catalytic degradation of simazine, with only a minimal loss in their catalytic activity (Boruah et al. 2021).

$$\begin{array}{c}{\text{FDG}}\left({{\text{Fe}}}_{3}{{\text{O}}}_{4}\right)+{\text{hU}}\to {{\text{Fe}}}_{3}{{\text{O}}}_{4}\left({{\text{h}}}^{+}\right)+{\text{DG}}\left({\text{e}}\right)\\ {\text{DG}}\left({\text{e}}\right)+{{\text{O}}}_{2}\to {\text{DG}}\left({\text{e}}\right)+{{\text{O}}}_{2}\\ \begin{array}{c}{{\text{O}}}_{2}+{{\text{H}}}_{2}{\text{O}}\to {{\text{HO}}}_{2}+-{\text{OH}}\\ {\text{DG}}\left({\text{e}}\right)+{{\text{HO}}}_{2}+{{\text{H}}}^{+}\to {{\text{H}}}_{2}{{\text{O}}}_{2}\\ \begin{array}{c}{\text{DG}}\left({\text{e}}\right)+{{\text{H}}}^{+}+{{\text{H}}}_{2}{{\text{O}}}_{2}\to {{\text{H}}}_{2}{\text{O}}+{\text{OH}}\\ {{\text{Fe}}}_{3}{{\text{O}}}_{4}\left({{\text{h}}}^{+}\right)+-{\text{OH}}\to {\text{OH}}+{{\text{Fe}}}_{3}{{\text{O}}}_{4}\\ {\text{Simazine}}+{\text{OH}}\to \left({{\text{CO}}}_{2}, {{\text{H}}}_{2},\mathrm{ CI},\mathrm{ and }{{\text{NO}}}_{3-}\right)\end{array}\end{array}\end{array}$$

Our focus will be directed towards the degradation and removal of organic pollutants using various nanozymes, as illustrated in Table 1.

**Table 1:** The degradation and removal of organic pollutants by different nanozyme

Nanozyme	Activity	Organic pollutant	Method	Synthesis method	References
CoFe_2_O_4_	Peroxidase	Degradation of Methylene blue dye and detection of H_2_O_2_	Colorimetric analysis	Phase transfer	(Wu et al. 2018; Feng et al. 2013)
Fe_3_O_4_@Gel	Peroxidase	Indigo carmine (IC)	Catalytic oxidation	Phase transfer	(Zha et al. 2022)
FeBi-NC dual active site	Oxidase	Rhodamine B and methyl orange		Pyrolysis	(Chen et al. 2022)
Au-NiFe layered double hydroxide (LDH)/rGO	Oxidase and peroxidase	Organic mercury and H_2_O_2_	Catalytic oxidation and formation of Au amalgam		(Liu et al. 2021b)
Pd nanozymes (PdNPs/PCNF)	Peroxidase and oxidase	Methylene blue	Production of ROS which degrade pollutant molecules	Vacuum filtration and freeze drying	(Dadigala et al. 2022)
MnO_2_^−^ and SiO_2_@Fe3O_4_^−^multinanozyme system	Peroxidase	Malachite green	Electron transfer	Co-precipitation	(Jangi et al. 2020)
Cu/H_3_BTC	Laccase	Amido black 10B dye and epinephrine			(Shams et al. 2019)
CuS–MoS_2_	Peroxidase such as natural horseradish peroxidase	Some chromogenic substrates like 3,3′,5,5′-tetramethylbenzidine, 2,2′-azino-bis (3-ethylbenzo-thiazoline-6-sulfonic acid) diammonium salt, and o-phenylenediamine			(Borthakur et al. 2021c)
CuS/g-C_3_N_4_ and CuS/h-BN	Peroxidase	Ibuprofen (Ibu)	Colorimetric analysis	Hydrothermal technique	(Borthakur et al. 2021b)
CH-CU	Laccase	Phenolic pollutants	Colorimetric analysis		(Wang et al. 2019)
FDGs	Peroxidase	simazine pesticide	Hydrogen bonding interaction		(Boruah et al. 2021)

### Fluorescent MOF-based nanozymes

Distinguishing between structural isomers of organic compounds has consistently posed a formidable challenge in environmental science due to their closely aligned physical and chemical characteristics, which arise from subtle variations in their molecular structures (Wang et al. 2013). o-Phenylenediamine (OPD), m-phenylenediamine (MPD), and p-phenylenediamine (PPD) are three isomeric forms of phenylenediamines that find applications as starting materials in a range of industries, including the manufacture of plastics, pharmaceuticals, and industrial dyes (Mathivanan et al. 2020; Shi et al. 2016). However, their damages to the human health and environment upon prolonged exposure are different. OPD is a highly toxic and carcinogenic environmental pollutant (Matsumoto et al. 2012; Nezamzadeh-Ejhieh & Salimi 2010). PPD can cause various immediate allergic reactions (Gu et al. 2019). MPD does not lead to obvious harmful effects.

#### Detection methods

While significant advancements have been achieved in the field of bioanalysis using peroxidase-like nanozymes, there is a noticeable scarcity of nanozyme-based biosensors designed to distinguish between isomeric organic compounds. In this study, we have harnessed the potential of a fluorescent metal–organic framework (MOF)-based nanozyme for the discrimination and detection of phenylenediamine isomers. Specifically, we have employed NH_2_-MIL-101(Fe), a member of the Fe-based MOFs, which not only serves as a fluorescent indicator but also mimics the activity of peroxidases. In the presence of hydrogen peroxide (H_2_O_2_), NH_2_-MIL-101(Fe) can catalyze the oxidation of both o-phenylenediamine (OPD) and p-phenylenediamine (PPD) into their respective oxidation products (OPDox and PPDox). Interestingly, this process results in the quenching of NH_2_-MIL-101(Fe)’s intrinsic fluorescence at 445 nm due to the inner filter effect (IFE). Conversely, a new fluorescence peak emerges at 574 nm for OPDox. Consequently, we have devised a ratiometric fluorescence method for the detection of OPD, utilizing the fluorescence intensity ratio *F*_*574*_*/F*_*445*_ as the readout. This innovative approach exhibits exceptional discriminatory capabilities for the three phenylenediamines and has the potential to open up new avenues for the utilization of MOFs in the field of environmental science (Xia et al. 2022).

### Covalent organic framework nanozyme for colorimetric detection of uranium

We have successfully synthesized a photosensitive covalent organic framework (COF) using porphyrin derivatives and the organic dye BDP as its constituent monomers, marking the first achievement of this kind. This COF, known as Tph-BDP, exhibits exceptional light-absorbing capabilities across a wide spectrum, ranging from ultraviolet to infrared, thanks to its extended π-conjugated structure. Moreover, due to its narrow energy level bandgap, Tph-BDP can rapidly generate O_2_^·−^ (superoxide radicals) when exposed to laser irradiation, facilitating the catalytic oxidation of TMB (3,3′,5,5′-tetramethylbenzidine). In the presence of even trace amounts of UO_2_^2+^ (uranyl ion), it forms complexes with the imines produced during the TMB oxidation, resulting in the precipitation of these complexes. This, in turn, hampers the charge transfer (CT) interaction between the donor and the acceptor, causing the characteristic blue color to fade. Consequently, the Tph-BDP-TMB colorimetric platform can effectively detect UO_2_^2+^ with high sensitivity and accuracy. Furthermore, this platform demonstrates excellent applicability in real water samples. This research lays a solid foundation for the development of photosensitive COFs and their utilization in the colorimetric detection of radionuclides (Zhang et al. 2021).

### Graphite-phase carbon nitride

It is the most stable allotrope of carbon nitride under ambient conditions (Gan et al. 2017). Graphitic carbon nitride (g-C3N4) is composed of carbon and nitrogen, both of which are readily available elements. It is known for its environmentally friendly and sustainable nature and can be manufactured on a large scale at a low cost. Notably, g-C3N4 possesses intrinsic peroxidase-like properties, making it suitable for the detection of substances such as H2O2 and glucose (Ma et al. 2014). The distinctive electronic, optical, structural, and physicochemical characteristics of g-C3N4 render it a remarkable material for various energy and environmental purposes, including the decomposition of organic contaminants. Nevertheless, when compared to natural peroxidases, the catalytic performance of g-C3N4 falls short of expectations. Hence, there is a critical need to develop exceptionally efficient nanozymes based on g-C3N4 (Ju et al. 2018).

The bandgap of g-C_3_N_4_ is approximately 2.7 eV, and it has conduction band (CB) and valence band (VB) potentials at approximately − 1.1 eV and 1.6 eV, respectively. This configuration enables it to undergo visible light-driven photocatalysis. However, pristine g-C_3_N_4_ has certain drawbacks, including a limited response range to visible light, low surface area, and inefficient separation of electron–hole pairs. These shortcomings collectively lead to a substantial reduction in its photocatalytic efficiency (Tan et al. 2021). In order to expand the utility of g-C3N4 in photocatalysis, a range of methods have been devised to enhance its capacity for light absorption and the efficient transfer of electron–hole pairs. These approaches encompass controlling its morphology, introducing defects, incorporating doping with different atoms, and forming associations with metal, semiconductors, and carbon-based materials.

As an illustration, creating a heterojunction represents a potent approach for enhancing photocatalytic performance. In this context, a composite material known as Mo, N co-doped ZnIn_2_S_4_/g-C_3_N_4_ (abbreviated as M, N-ZIS/CN), was synthesized using the sol–gel method for the purpose of degrading methylene blue through photocatalysis (Ma et al. 2022b, c, a).

Enhancing the photocatalytic performance of g-C_3_N_4_ can be effectively achieved through methods such as doping and the introduction of defects. In this case, modified g-C_3_N_4_ was created via thermal polymerization, utilizing urea as the primary raw material and EDTA-2Na as a modifier, with the aim of degrading bisphenol A (BPA) through photocatalysis (He et al. 2022).

### Phenols

In the environment, a wide array of organic contaminants exists, and numerous among them pose challenges when it comes to their detection. Nanozymes present a viable solution for identifying and dismantling various harmful chemical substances, such as pesticides and phenolic compounds (Xu et al. 2021).

Laccases are multi-copper oxidases that can reduce molecular oxygen to water while oxidizing a variety of substances, including substituted phenols and aromatic amines. For these practical applications, laccase-like nanozymes are a possible replacement for laccase due to their excellent stability. The complicated structure of laccase’s active site and catalytic mechanism may account for the paucity of studies on nanozymes exhibiting laccase-like activity (Gugoasa et al. 2021). Laccase (EC 1.10.3.2), functioning as a multi-copper oxidase (MCO), possesses the ability to facilitate the oxidation of diverse organic substrates, with a particular emphasis on polyamines and phenolic compounds. This enzymatic process employs oxygen as the primary oxidant, resulting in the production of water rather than H2O2. Leveraging the alteration in color induced by the oxidation of phenols, laccase-based sensors can effectively and swiftly identify phenolic substrates through colorimetric and electrochemical assays (Jiang et al. 2018).

It has also been investigated if carbon dots (CD-based nanozymes) can be used as catalysts for the elimination of phenolic chemicals compared with the effectiveness of a CD-based nanozyme (GQDs/Fe_3_O_4_) and horseradish peroxidase (HRP) at removing phenolic chemicals. According to the results, GQDs/Fe_3_O_4_ were more effective at removing phenol than HRP. The elimination effectiveness of GQDs/Fe_3_O_4_ was lower to equivalent to HRP for the other phenolic compounds examined. The synthesized GQDs/Fe_3_O_4_ exhibits several advantages compared to HRP, thereby demonstrating its potential for wastewater treatments. Notably, it possesses high thermal stability, making it resilient under elevated temperatures. Additionally, it is cost-effectiveness as it can be produced at a lower expense. Moreover, the GQDs/Fe_3_O_4_ composite demonstrates reusability, enabling its repeated application in wastewater treatment processes (Wu et al. 2019).

#### ***Enhanced peroxidase-like activity of MoS***_***2***_***-Pt***_***3***_***Au***_***1***_*** nanocomposites for colorimetric phenol detection***

Cai et al. conducted that MoS_2_-based nanocomposites have emerged as versatile and groundbreaking materials. Cai et al. achieved the synthesis of nanocomposites by embellishing a few-layer MoS_2_ nanosheet matrix with Pt_3_Au_1_ nanoparticles (NPs), introducing a fresh category of enzyme mimics. Impressively, these MoS_2_-Pt_3_Au_1_ nanocomposites exhibited a substantially heightened peroxidase-like activity relative to unmodified MoS_2_ nanosheets. Furthermore, these nanocomposites demonstrated excellent dispersibility in water and exceptional stability. Building on these outcomes, they established a straightforward, expeditious, and cost-effective colorimetric technique for the precise and selective detection of phenol. This method relies on the oxidative coupling reaction between phenol and 4-aminoantipyine in the presence of H_2_O_2_ as an oxidizing agent, resulting in the generation of pink-colored reaction products (Cai et al. 2017).

Table 2 provides a comprehensive overview of various nanozymes and their activities in the detection and degradation of different phenolic compounds. It showcases a wide range of nanozymes, including their enzyme-like activities, targeted pollutants, synthesis methods, functions, and relevant references. This compilation is a valuable resource for researchers and professionals in the field of environmental science and nanotechnology, as it succinctly presents key information on nanozyme applications for phenol-related environmental challenges. Well organized and informative, this table serves as a useful reference for understanding the diverse capabilities of nanozymes in addressing phenol pollution.

**Table 2:** Detection and degradation of several phenols by different nanozymes

Nanozyme	Enzyme-like activity	Pollutant	Synthesis method	Function	Limit of detection	References
Cp-cu	Laccase	o-aminophenol hydroquinone	Hydrothermal method	Degradation	/	(Xu et al. 2021)
Au-rgo nps	Laccase	Catechol	Exfoliating graphite rods	Detection by electrochemical sensor	3.3 × 10^−6^ M	(Gugoasa et al. 2021)
Amp-cu	Catalytic activity	Phenols in orange juice	Biotechnology method	Detection	0.033 μmol·L^−1^	(Huang et al. 2021)
Cu-MIM	Laccase	Wide range of Phenols	Self-assembly of Cu ions with various IM derivatives	Sensitive detection and degradation	3σ/b	(Lei et al. 2022)
Mnp@cts	Peroxidase	Wide range of phenols	Hydrothermal method with self-assembly	Decomposing and degredation	/	(Jiang et al. 2018)
Mnco@C ncs	Laccase & catalytic activity	2,4-DP	One-step calcination of polydopamine-coated mnco Prussian blue analogs (mnco-PBA@PDA)	Detection by colorimetric sensor	0.0778–0.218 μM	(Zhu et al. 2022a, b)
Nife_2_o_4_	Peroxidase	2,4-DP	Co-precipitation method using ferric chloride and nickel chloride hexahydrate	Detection by colorimetric method	0.311 µg·mL^−1^	(Omar and Jabbar 2022)
2H–mos_2_/Co_3_O_4_	Oxidase & catalytic activity	2,4,6-trinitrotoluene	In-situ growth method by uniform and subvertical assembly of ultra-thin Co_3_O_4_ nanoflakes onto 2H–mos_2_ nanosheets	Detection by electrochemical microsensor	1 pM	(Ma et al. 2022a)
Nico_2_o_4_@mno_2_	Peroxidase &Oxidase	Hydroquinone	Nico_2_o_4_@mno_2_ microcubes with p–n junctions	Detection by colorimetric method	0.042 μM	(Ma et al. 2022b)
Co1.5mn1.5o_4_	Laccase, peroxidase, oxidase, and catalase	Hydroquinone	Constructing a metal–organic framework by doping manganese ions into fe-mof by introducing bimetallic active centers	Detection by colorimetric sensor	0.04 μM	(Liu et al. 2021c, d, e, f, a, b)
Fe_3_O_4_@COF	Peroxidase	Hydroquinone	One-pot assembly procedure	Detection by electrochemical enzymatic biosensor	0.12 μmol L^−1^	(Sun et al. 2022)
BSA-Cu laccase mimicking	Peroxidase & oxidase	Catechol, dopamine, guaiacol, resorcinol, 4-phenylazophenol	Self-assembly of BSA & cuso4	Detection & degradation	0.38 µg/mL	(Huang et al. 2022b, a)
Fe1@CN-20	Laccase	P-chlorophenol, 2,6-dimethoxyphenol, 2,4-DP, catechol	Chemical procedure	Sensitive detection & degradation by oxidation	1.3 µM	(Lin et al. 2022)
Amorphous imidazole-Cu	Oxidase & catecholase & laccase	2,4-DP, hydroquinone, catechol and 2,6-dimethoxyphenol	Water induced precipitation of Cu^2+^ and imidazole	Oxidation and detection	0.412 μM	(Wang et al. 2022a)
Bioinspired CH-Cu	Laccase, catalytic activity	Chlorophenols and bisphenols	Aqueous Cys-His dipeptide solution & aqueous cucl2	Degradation & detection	0.31 μg/mL	(Wang et al. 2019)
Fe_3_o_4_@cu/gmp	Laccase	O-phenylenediamine & phenolic compounds	Coprecipitation of fecl_3_·6H_2_o and fecl_2_·4H_2_o	Degradation & oxidation	/	(Zhang et al. 2020a, b)
Amorphous MOF	Catecholase & laccase	2,4-DP, hydroquinone, catechol and o-aminophenol	Facile solvothermal reaction	Degradation & detection	0.889–0.990 μM	(Wang et al. 2022b)
Cu-Cys@COF-ome	Laccase	Benzoquinones	Ultrasonic dissolving	Degradation	/	(Tang et al. 2022)
Gqds	Superoxide dismutase	2,4-DP, hydroquinone	Chemical oxidation	Detection & degradation	0.01–0.1 μM	(Wu et al. 2019)
N-cds and manganese oxide/ferric oxide hybrids	Peroxidase	Wide range of Phenols	Hydrothermal	Detection & Elimination	84 nM	(Ngo et al. 2021)

Our emphasis will be on the detection and degradation of phenols using a variety of nanozymes, as illustrated in Table 2.

#### Toxic ions

In the contemporary era, the safety of human life and health faces a severe threat from environmental contamination. In recent decades, incidents of inorganic ion pollution, notably involving Cl^−^ and Hg^2+^, have been reported across a vast expanse of global regions. Owing to its non-biodegradable nature and its distinctive affinity for sulfur-containing ligands in humans and other species, even minute quantities of Hg^2+^ can induce a range of disorders in the central nervous system, the human kidney, and the brain (Vanjare et al. 2021). As a significant bio-electrolyte, Cl^−^ is widely distributed within cells and plays a crucial role in a multitude of physiological activities. Nevertheless, cystic fibrosis, startle reactions, and myotonia diseases are often associated with disturbances in this homeostatic regulation within bodily fluids, including sweat (Zhao et al. 2019).

### *Hg*^*2*+^*and Cl*^*−*^

#### AgRu bimetal mesoporous nanozyme

β-Cyclodextrin and co-enhanced graphene oxide AgRu bimetallic mesoporous nanozymes have been utilized for the detection and removal of Hg^2+^ and Cl^−^. The application of diverse nanozymes facilitated the detection and elimination of Hg^2+^ ions, as demonstrated in Table 3.

**Table 3:** The detection and removal of Hg^2+^ by different nanozyme

Nanozymes	Enzyme mimic activity	Methods	References
Silver nanoparticles	Peroxidase	O^−^phenylenediamine/H_2_O_2_ system for fluorescence detection	(Abdel-Lateef 2022)
L-Cysteine functionalized grapheme oxide nanoarchitectonics	Peroxidase	Competitive adsorption	(Tian et al. 2022)
Porous cerium oxide nanorod	Peroxidase	Fluorescence/colorimetry dual-mode sensing strategy	(Li et al. 2022b)
Au nanoparticle-hydrogel nanozyme	Peroxidase	Colorimetric detection	(Ko et al. 2021)
CuS and NiS nanoparticle-decorated porous-reduced graphene oxide sheets	Peroxidase	Colorimetric detection	(Borthakur et al. 2021a)
A strongly coupled Au/Fe_3_O_4_/GO hybrid material	Peroxidase	Colorimetric detection, and removal of Hg^2+^ in aqueous solutions	(Zhang et al. 2015)

**Fig. 1:**
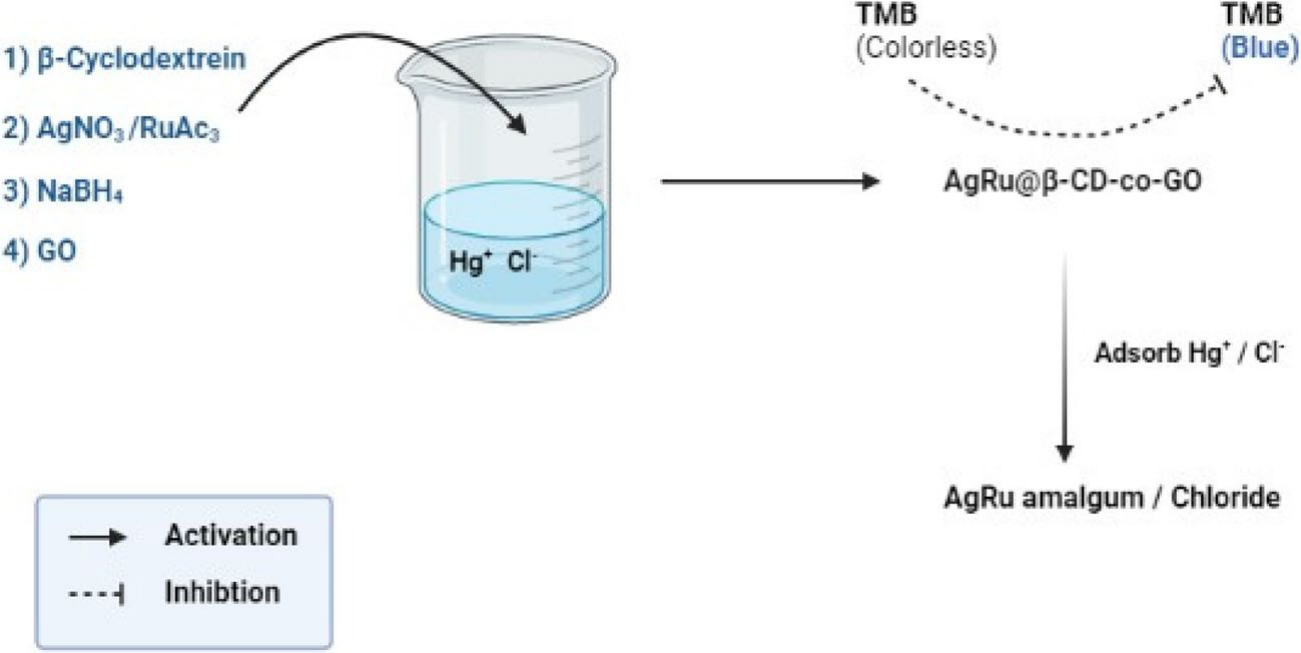
Mechanism of action of AgRu@β-CD-co-GO

The AgRu bimetallic mesoporous nanozyme, denoted as AgRu@β-CD-co-GO, was synthesized using a conventional redox method. Due to its porous microstructure and the presence of various functional groups, such as GO aryl rings and hydroxyls, the synthesized AgRu@β-CD-co-GO exhibited notable peroxidase-like activity and exceptional adsorption capability for enrichment. In the presence of H_2_O_2_, AgRu@β-CD-co-GO effectively catalyzed the conversion of colorless TMB into blue oxTMB. Incorporating layers of AgRu chloride or AgRu amalgam onto the surface of AgRu@β-CD-co-GO hindered the peroxidase-like activity in the presence of toxic Cl^−^ and Hg^2+^ ions, as depicted in Fig. 1. Under optimal conditions, AgRu@β-CD-co-GO was successfully employed for colorimetric detection and enhanced elimination of toxic Hg^2+^ and Cl^−^ ions in real samples, resulting in a distinct color change from bright blue to colorless (Yan et al. 2022).

#### ***Removal of Hg***^***2***+^***and Cl***^***−***^*** by simple filtration***

The ability of AgRu@β-CD-co-GO to effectively enrich and separate Hg^2+^ and Cl^−^ ions was assessed, considering its unique interaction with these toxic species, as well as its porous structure and high specific surface area. To evaluate this, different quantities of standard Hg^2+^ and Cl^−^ samples were introduced into the AgRu@β-CD-co-GO-H_2_O_2_-TMB system. The resulting mixtures were vigorously stirred for a duration of 30.00 min, followed by filtration through commercial microporous filter sheets with a pore size of 0.22 µm, repeated three times. Subsequent analysis revealed that the removal of Hg^2+^ and Cl^−^ ions could be achieved simply by filtration through these commercially available microporous films, with removal efficiencies exceeding 95.4% and 93.8%, respectively (Yan et al. 2022), Hg^2+^ was detected and removed as shown in Table 3.

### Copper ion

The functions of vital organs in the human body, including the liver, heart, and the brain, depend on copper ions (Cu^2+^), essential microelements. However, excessive intake of Cu^2+^ can be toxic to humans, and high levels of Cu^2+^ also have deleterious effects on the environment. Accumulation of Cu^2+^ likely contributes to the production of reactive oxygen species, potentially leading to neurodegenerative disorders. Cu^2+^ has also been associated with a group of other diseases, including liver cirrhosis with ascites, cardiovascular disease, atherosclerosis, cancer, and diabetes (Liang-Liang et al. 2017).

#### Mechanism of action

A sensitive colorimetric method for detecting copper ions has been developed by modifying the surface of Ag/Pt nanoclusters (Ag/Pt NCs) and controlling their peroxide-like activity. It was observed that the catalytic activity of Ag/Pt NCs was hindered by the presence of (MPA) 3-mercaptopropionic acid; however, this inhibitory effect was eliminated upon oxidation by oxygen facilitated by Cu^2+^ catalysis. By monitoring the changes in the colorimetric signal of the TMB-H_2_O_2_ reaction, a colorimetric approach for detecting Cu^2+^ was devised. With a detection limit of 5.0 nM, this method exhibited higher sensitivity and selectivity for Cu^2+^ compared to other metal ions. Furthermore, this technique was user-friendly, cost-effective, and suitable for the detection of Cu^2+^ in real water samples (Liang-Liang et al. 2017).

### Ferrous ion

In both industry and agriculture, the iron ion is widely utilized and is abundant throughout the universe. Numerous environmental processes depend on this element. Consequently, methods for the rapid and facile detection of ferrous ions (Fe^2+^) in environmental samples, especially water samples, are of paramount importance (Hu et al. 2016).

#### Mechanism of action

**Fig. 2:**
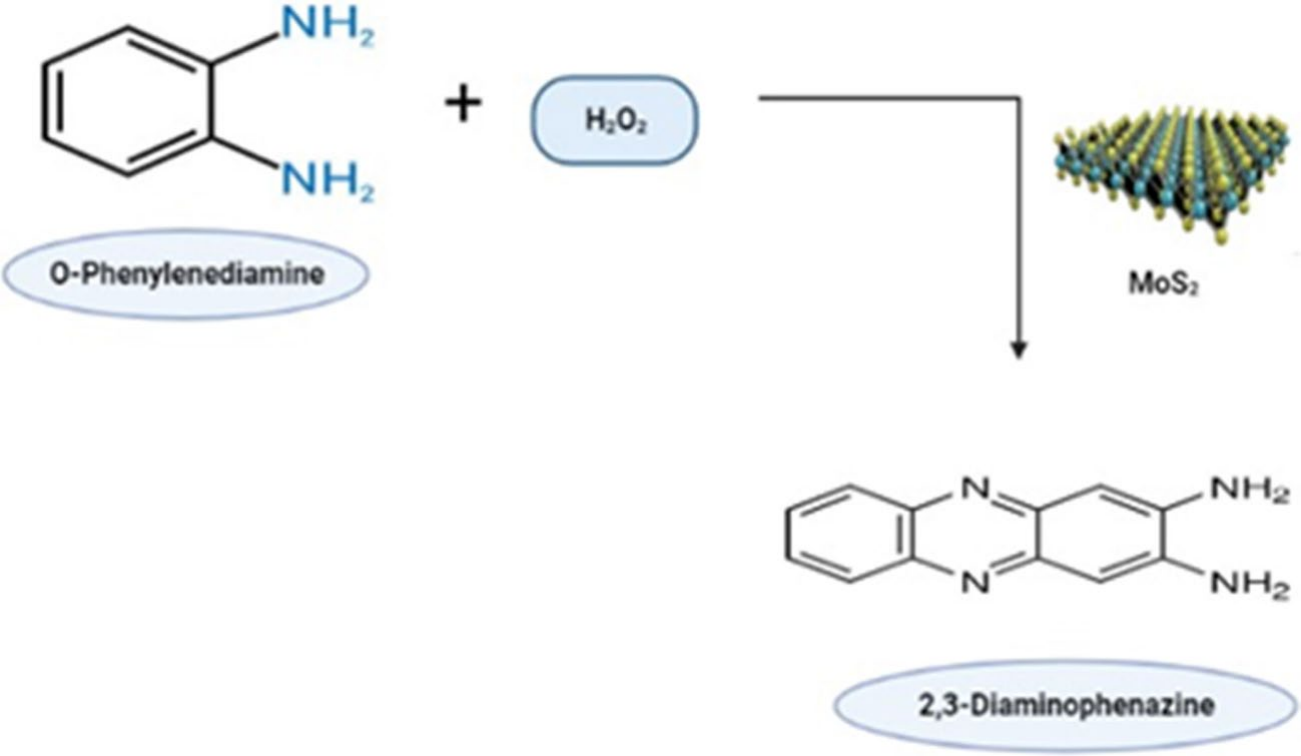
The action of MoS_2_ mimic peroxidase enzyme

The layered MoS_2_ nanoflares, produced via the hydrothermal technique, can catalyze the oxidation of o-phenylenediamine (OPD) substrate by H_2_O_2_ to form the highly fluorescent compound 2,3-diaminophenazine (DAPN), thus confirming the capacity of MoS_2_ nanosheets to serve as peroxidase mimics, as depicted in Fig. 2. In the presence of Fe^2+^, the catalytic activity of MoS_2_ nanosheets is significantly augmented, surpassing their standalone performance. Consequently, MoS_2_ nanosheets were employed as a novel type of fluorescent catalytic biosensor for the detection of Fe^2+^ through the OPD/H_2_O_2_ catalyzed fluorescence mechanism. Furthermore, it was observed that the utilization of MoS_2_ nanosheets can enhance both the sensitivity and selectivity of the fluorescent catalytic biosensors. The developed MoS_2_/OPD/H_2_O_2_ biosensing technology exhibited remarkable sensitivity and selectivity in Fe^2+^ detection, with a limit of detection of 3.5 nM and an analytical range spanning from 0.005 to 0.20 M (Hu et al. 2016).

### Cyanide ion

The human body exhibits a high sensitivity to cyanide ions (CN^−^). A substantial binding of CN^−^ to one of the heme units of cytochrome c oxidase effectively halts cellular respiration, leading to severe damage to the central nervous system. In cases of extreme CN^−^ exposure, human mortality can occur within mere minutes. The lethal oral dose of hydrogen cyanide for humans is approximately 1.4 mg/kg of body weight (1.4 mg kg^−1^) (Lien et al. 2018).

#### Mechanism of action

**Fig. 3:**
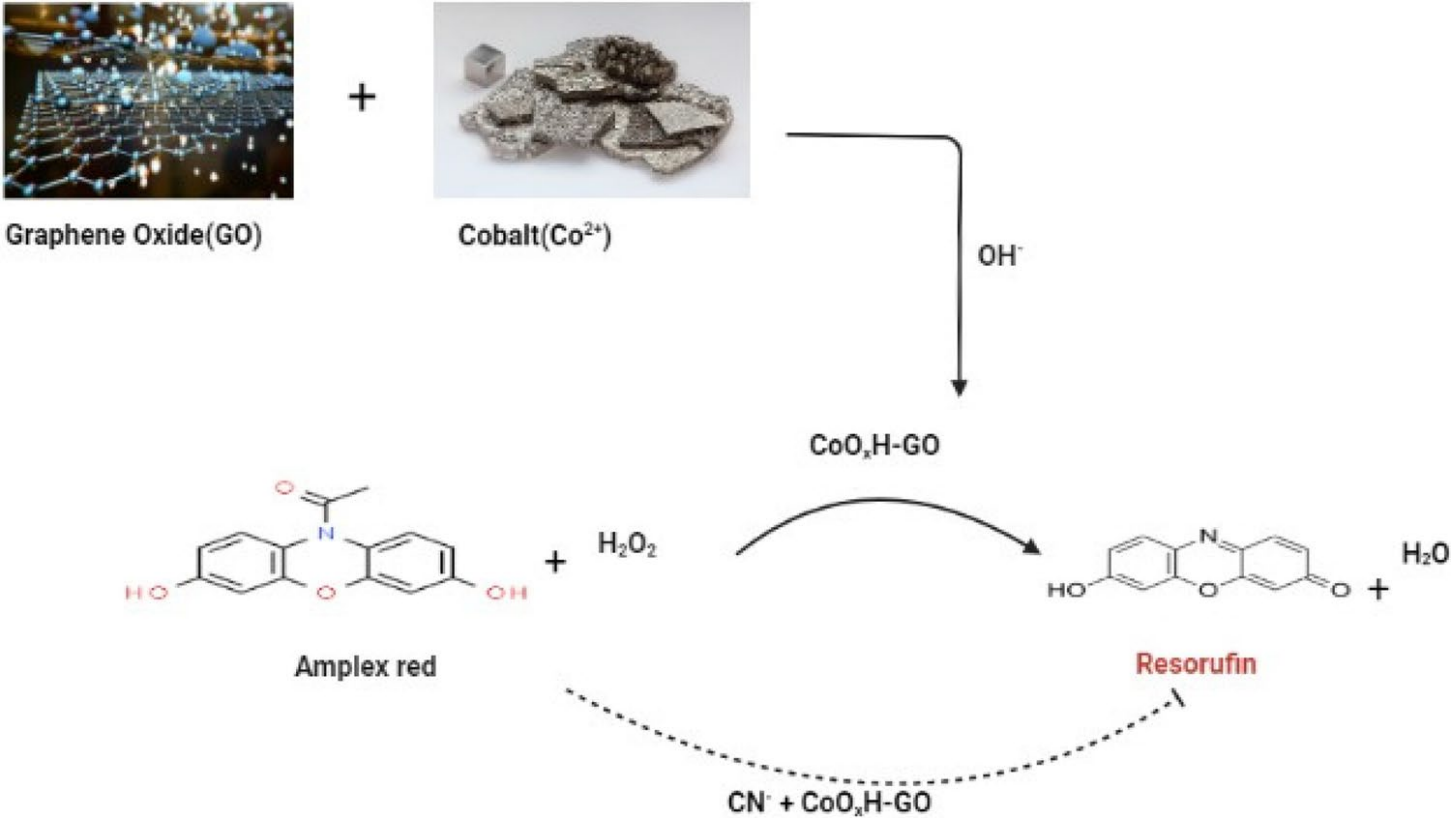
The cyanide’s effect on (CoOxH-GO) nanohybrid’s catalytic activity

A nanohybrid material, denoted as CoOxH-GO (comprising amorphous CoOxH-modified graphene oxide), was synthesized through a straightforward reaction between graphene oxide (GO) and Co^2+^ in an alkaline solution. In comparison to CoOxH (cobalt hydroxide/oxide) and GO in isolation, CoOxH-GO displayed enhanced peroxidase mimic activity for the oxidation of Amplex red (AR) by H_2_O_2_. The AR/CoOxH-GO probe exhibited a notably low limit of detection (LOD) at 32 nM, with an expansive linear range spanning from 100 nM to 100 M for H_2_O_2_ detection. It is worth noting that the catalytic performance of CoOxH-GO was compromised due to the formation of the Co(II)-CN complex, which hindered the electron transfer from CoOxH-GO to the substrate, as illustrated in Fig. 3. For CN^−^ detection, the H2O2/AR-CoOxH-GO probe achieved an LOD of 22 nM and demonstrated exceptional selectivity (> 100-fold) towards other anions. Furthermore, in the presence of AR and H2O2, CoOxH-GO/N^+^M facilitated the production of a reddish resorufin product, and CN^−^ effectively inhibited this product, enabling the visual detection of CN^−^ at the nanomolar level. These advantageous characteristics enabled the detection of CN^−^ in high-salinity seawater without interference from highly concentrated background ions (Lien et al. 2018).

### Fluoride ion

This fluoride ion holds significant importance in numerous applications (Zhou et al. 2014). For instance, it is incorporated into toothpaste for the purpose of demineralizing and remineralizing teeth (Oh et al. 2017). In certain bacterial cells, a riboswitch exists to regulate the intracellular concentration of fluoride (Baker et al. 2012). Nevertheless, elevated concentrations of fluoride can also be toxic, leading to conditions such as bone fractures, urolithiasis, and cancer (Everett 2011), and it is important to measure fluoride in various samples. Fluorine possesses numerous distinctive chemical properties, being the element with the highest electronegativity. The adsorption or incorporation of the highly electronegative F^−^ ion can effectively modify the energy bands of nanomaterials. These attributes hold promise for applications in its detection (Ahmad et al. 2014; Karlický et al. 2013).

#### ***Colorimetric and fluorescence detection methods of F***^***−***^

In recent times, the exploration of utilizing nanomaterials with enzyme-mimicking capabilities for the development of biosensors has gained attention. A recent report highlighted the substantial enhancement of the oxidase-like activity of CeO_2_ nanoparticles in the presence of fluoride. This advancement enabled the demonstration of colorimetric fluoride detection using 2,2′-azino-bis(3-ethylbenzothiazoline-6-sulphonic acid) (ABTS) as a substrate (D. Li et al. 2019a, b, c). Fluoride is used to boost the activity of nanoceria as an oxidase mimic (Liu et al. 2016). In this reaction, the detection method relied on observing the color change in ABTS following oxidation. However, it is crucial to explore the possibility of detecting fluoride using a fluorescence signal. This approach holds the potential to enhance signaling sensitivity and opens up avenues for various applications, including intracellular detection. In oxidative reactions, one of the commonly employed substrates is Amplex red (AR), which, upon oxidation, generates the red fluorescent compound resorufin. The oxidation of AR by CeO_2_ nanoparticles is notably influenced by both pH and fluoride concentration. Both acidic conditions and the presence of fluoride can significantly enhance the oxidation activity of CeO_2_ nanoparticles, occasionally leading to excessive oxidation resulting in the less fluorescent compound resazurin. Under optimized conditions, specifically at pH 4.0 with a reaction time of 2 min, fluoride was detected at concentrations as low as 1.8 μM, exhibiting exceptional specificity. It is worth noting that only phosphate showed interference, but at environmentally relevant concentration levels, phosphate is unlikely to pose a concern. Among the various metal oxide nanoparticles tested, only CeO_2_ demonstrated enhanced activity in the presence of fluoride. Therefore, this reaction could also potentially be utilized for the detection of CeO_2_ (D. Li et al. 2019a, b, c).

#### The effect of fluoride ion on nanozyme

R-MnCo_2_O_4_/Au nanotubes have been successfully synthesized and exhibit exceptional peroxidase-like activity, rendering them an effective substrate for surface-enhanced Raman spectroscopy (SERS). In the realm of efficient SERS substrates, these R-MnCo_2_O_4_/Au nanotubes have demonstrated their capability to detect CV^+^ probes, achieving a remarkable sensitivity even at concentrations as low as 0.1 μM. Additionally, these RMnCo_2_O_4_/Au nanotubes possess the ability to catalyze the oxidation of a TMB system and replicate the inhibitory effect of the system upon the introduction of F^−^ ions. Further investigation into the mechanism behind the inhibition of the catalytic peroxidase-like reaction has revealed that it involves the suppression of the charge transfer process and the inhibition of reactive oxygen species (ROS) generation. These findings suggest that the formation of Fe − F bonds through the combination of F^−^ ions with Fe^3+^ plays a pivotal role in the inhibition effect within this novel nanozyme–SERS system. As a result, this system offers highly sensitive detection capabilities for F^−^ (Wen et al. 2020).

### Chromium ion

Chromium (VI) is a heavy metal ion characterized by its considerable solubility, mobility, resistance to biodegradation, potent carcinogenic properties, and toxicity levels approximately 100 times greater than those of chromium (III) (Kim et al. 2019; Nghia et al. 2018). The US Environmental Protection Agency (EPA) establishes a maximum allowable concentration of chromium (VI) in drinking water at 1 μM (50 ppm) (Guo et al. 2016; S. Liu et al. 2021c, d, e, f, a, b).

#### Detection method of chromium by nanozyme

A unique nanocomposite, CoFe_2_O_4_/H_2_PPOP, consisting of organic–inorganic components, was synthesized through the in situ deposition of CoFe_2_O_4_ nanocubes onto a fully conjugated porphyrin-based porous organic polymer (H_2_PPOP). CoFe_2_O_4_/H_2_PPOP exhibited exceptional tetraenzyme-like activities, encompassing oxidase-like, peroxidase-like, catalase-like, and superoxide dismutase-like activities. In comparison to pure CoFe_2_O_4_ nanocubes, the catalytic capabilities of CoFe_2_O_4_/H_2_PPOP were significantly enhanced due to the substantial surface area and extended conjugated structure of H_2_PPOP, the presence of abundant active substances (CoFe_2_O_4_) on the surface, and the efficient electronic transfer between CoFe_2_O_4_ and H_2_PPOP. Leveraging the oxidase-like activity of CoFe_2_O_4_/H_2_PPOP, a colorimetric platform was established for the detection of chromium (VI) (Cr (VI)) with a broad linear range spanning from 0.6 to 100 μM and a low detection limit of 26 nM. Furthermore, by harnessing the dual oxidase-like and peroxidase-like activities, a more sensitive colorimetric platform for Cr (VI) was developed, offering faster detection speed and a remarkably low limit of detection (2 nM). This research introduces a novel approach to fabricate multi-enzyme active nanozymes and explores their potential for the detection of environmental pollutants (Guo et al. 2022).

### Sulfide ion

Sulfide ions, considered toxic contaminants, can be encountered in both natural water sources and wastewater. They often appear as by-products resulting from various industrial processes, including food manufacturing, tanneries, and petroleum refining. Exposure to high concentrations of sulfide ions can lead to severe health issues in humans, including Alzheimer’s disease, Down syndrome, and hyperglycemia (Liu et al. 2020).

#### Detection mechanism of sulfide ion

We have devised a smart system involving casein/PtNP nanozymes with peroxidase-mimicking capabilities and explored the modulation of CM-PtNP nanozyme activity induced by sulfide ions. Interestingly, we observed that sulfide ions can elicit distinct switching effects on the activity of casein/PtNP depending on the substrate used and the surface structure of the nanozyme, which can be precisely tuned. We delved into the mechanism of this switching phenomenon through a comprehensive analysis involving XPS, TEM, fluorescence spectra, and FTIR studies. Our findings indicate that S^2−^ ions deactivate the nanozyme by obstructing active sites and activate it by reducing Pt^2+^ ions to Pt0 species. Leveraging this unique responsiveness, we developed a colorimetric sensor for sulfide ions based on CM-PtNP. This sensor allows for the detection of S^2−^ ions with remarkable sensitivity, achieving a detection limit of 5 nM when employing TMB as the substrate and an even lower limit of 0.8 nM when using ABTS as the substrate (Liu et al. 2017).

## Pesticides

Pesticides play a crucial role in agriculture, significantly improving crop yield and quality. However, a major concern is the poor degradability of many pesticides, leading to their persistence in agricultural products and ecosystems. This persistence poses significant risks to both human health and the environment. Organophosphates serve as a prime example of such pesticides (Damalas & Eleftherohorinos 2011).

Organophosphate pesticides (OPs) represent a prominent category of commercially accessible pesticides. They find extensive application in contemporary commercial sectors for pest management and crop maintenance in agriculture (Eddleston et al. 2008).

**Fig. 4:**
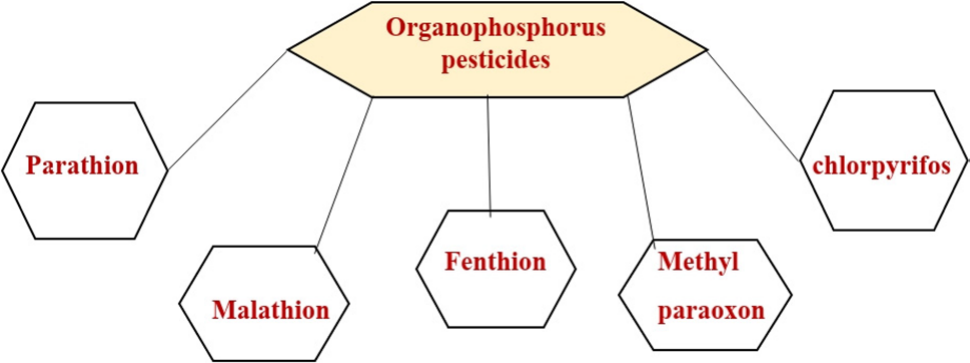
The commonly used organophosphorus pesticides

Organophosphates (OPs) act as potent neurotoxic compounds by functioning as irreversible inhibitors of the enzyme acetylcholinesterase (AChE). This interaction takes place through the binding of OPs to the active site of AChE, impacting a range of organisms, including insects, among others (Jokanović, 2018). The dysfunction of acetylcholinesterase (AChE) functionality results in the accumulation of the neurotransmitter acetylcholine, leading to excessive synaptic transmission, a factor implicated in human neurodegenerative diseases. Consequently, the widespread use of these pesticides raises substantial concerns regarding environmental residues stemming from improper application and subsequent entry into the food chain. These residues have the potential to cause significant harm to human health. Hence, there is an ongoing imperative to devise proficient methodologies and tools for the measurement and surveillance of OPs, as these endeavors play a pivotal role in addressing global health issues (L. Huang et al. 2019a, b). We will present examples of commonly used OPs, as depicted in (Fig. 4).

Gas chromatography (GC), high-performance liquid chromatography (HPLC), and capillary electrophoresis are widely utilized techniques in pesticide detection. These methods incorporate various detectors to ensure precise and dependable pesticide identification (Wei et al. 2016). The methods employed by these instruments exhibit remarkable characteristics such as enhanced sensitivity, selectivity, and precision. Nonetheless, their application requires highly skilled personnel and involves intricate sample preparation processes, which entail complex manufacturing steps and substantial usage of organic solvents (L. Huang et al. 2019a, b).

Over the past few decades, a variety of synthetic enzymes known as nanozymes have emerged, showcasing catalytic capabilities that can match or even exceed those of natural enzymes. Significantly, these nanozymes address constraints related to inadequate durability and suboptimal performance under various environmental circumstances (Wei & Wang 2013). High stability, catalytic, and easy to manufacture nanozymes are established rapid verification procedure (Y. Huang et al. 2019a, b).

Currently, nanozymes investigated for pesticide analysis fall into two categories: oxidoreductases and hydrolases. Oxidoreductases, commonly employed in pesticide detection, encompass oxidases and peroxidases. Their primary role is to furnish catalytic amplification signals by interacting with specific substrates. For instance, Fe_3_O_4_ magnetic nanoparticles (MNPs) exhibit horseradish peroxidase (HRP)-like activity. They can facilitate the oxidation of compounds such as 3,3′,5,5′-tetramethylbenzidine (TMB), 3,3′-diaminobenzidine (DAB), o-phenylenediamine (OPD), and 2,2-azobis(3-ethylbenzothiazoline-6-sulfonic acid) (ABTS) in the presence of H_2_O_2_, resulting in the generation of colored and fluorescent products (Gao et al. 2007).

On the other hand, in nanozyme-based pesticide testing, phosphatase mimetics serve as hydrolases. These phosphatase-like nanozymes provide quantitative data by hydrolyzing organophosphates (OPs) (Liu & Liu 2020).

The assays are based on phosphatase-like nanozymes:

In addition to the well-known peroxidase and oxidase-like catalytic functions of nanozymes utilized in pesticide detection, nanozymes exhibiting phosphatase-like activities have also been identified. These nanozymes can cleave the phosphate ester bond found in organophosphates (OPs). In such assays, OPs serve a dual role as both analytes and reagents, contributing to signal generation. Prominent examples of phosphatase-like nanozymes encompass materials such as cerium (Ce) and zirconium (Zr) (Mondloch et al. 2015). As an illustration of pesticide identification, Wu and colleagues designed a ZrO_2_/CeO_2_/PAA nanocomposite exhibiting impressive phosphatase mimicry. This nanocomposite effectively degrades methyl-paraoxon, leading to the formation of yellow p-nitrophenol, enabling colorimetric detection. Additionally, by leveraging the internal filtration effect involving p-nitrophenol and carbon nanodots, this approach allows for the fluorescence-based detection of the target methyl-paraoxon (J. Wei et al. 2019a, b). Moreover, Sun and colleagues accomplished electrochemical determination of the target by effectively monitoring the oxidation flux of p-nitrophenol produced during the hydrolysis of methyl-paraoxon catalyzed by CeO_2_ as a phosphatase mimic (Sun et al. 2021).

### Cerium oxide

Cerium oxide nanocrystals (nanoceria) exhibit an unusual mixed valence derived from the two oxidation states between Ce^4+^ and Ce^3+^ (Asati et al. 2009). Nanoceria is commonly recognized as nanozymes owing to their ability to catalyze specific biologically significant reactions, resembling the performance of conventional enzymes (Shin et al. 2015). The unique electronic configuration of cerium oxide grants it remarkable catalytic activity, particularly in redox reactions. Subsequently, extensive scientific inquiry has been devoted to exploring the OXD mimetic, catalase mimetic, peroxidase mimetic, and superoxide dismutase mimetic properties of cerium oxide (G. Chen et al. 2019a, b; Roushani & Shahdost-fard 2016).

### Platinum NPs

Pesticides can bind to platinum nanoparticles (Pt NPs) through different functional groups, leading to interactions that can modify the accessibility of active sites, ultimately affecting the concurrent modulation of Pt NPs’ catalytic activity (Zhu et al. 2022a, b).

### Manganese dioxide

The unique catalytic properties exhibited by manganese dioxide (MnO_2_), such as its oxidase and peroxidase-like activity, make it exceptionally well suited for integration into a wide range of sensing systems intended for the detection of pesticides (Wang et al. 2011).

### Graphene oxide

Graphene, a single atomic layer allotrope of graphite, boasts numerous exceptional attributes. These include its significant intrinsic surface area, high electron mobility, exceptional optical transparency, mechanical flexibility, and the potential for functionalization. When combined with semiconductors, graphene enhances photocatalytic activity. Additionally, graphene oxide (GO) exhibits peroxidase-mimicking activity, which is employed in pesticide detection and enhances photocatalytic efficiency (Boruah et al. 2021).

Presently, the majority of pesticide detection methodologies rely on integrated bioenzyme-nanozyme assays. These assays typically involve ternary or binary systems, wherein natural enzymes like ChE serve as recognition units, and nanozymes can only partly substitute these biological counterparts (e.g., HRP). Because these assays still employ relatively fragile natural enzymes, their susceptibility to harsh conditions poses significant challenges in practical applications. To address this issue effectively, a viable approach involves immobilizing biological enzymes and nanozymes together on suitable substrates (Jin et al. 2019). Our emphasis will be on the detection of various pesticides using various methods (Table 4).

**Table 4:** The different methods for pesticide detection

Real sample	Catalytic activity	Analyte	Detection mechanism	Testing method	References
Water & medical plants	Phosphatase	Methyl paraoxon	IFE-based fluorescence detection	Ceo_2_/carbondotes	(Wei et al. 2019a, b)
Cabbage	Peroxidase	Chlorpyrifos	Colorimetric detection	3D porous CeGONR	(Lin et al. 2020b, a)
Herbal plant	Organophosphorus hydrolase	Methyl paraoxon	Electrochemical detection	Ceo_2_ modified GCE	(Sun et al. 2021)
Apple skin	Peroxidase	Malathion & glyphosate	Colorimetric detection	TMB-Pt NPs multichannel colorimetric sensor array	(Li et al. 2022a)
Water, pear, cabbage, and rice	Catalyze substrate for signal amplification & peroxidase	Parathion	Colorimetric & fluorescence detection	Sensitive bio-barcode immunoassay based on bimetallic Au@Pt	(Chen et al. 2021)
Chinese cabbage	Oxidase	Ops pesticides	Colorimetric Detection	Portable sensor based on the reaction of ACHE-(γ- MnooH NWs) nanozyme- TMB system	(Huang et al. 2019a, b)
Rice & wheat	Oxidase	Fenthion	Chromatographic (colorimetric detection)	Enzyme nanozyme integrated hierarchically porous hydrogel (AChE-MnO_2_@HPH) for the smartphone-assisted convenient	(Zhu et al. 2022a, b)
Water sample	Peroxidase	Atrazine	Colorimetric Detection	Magnetic Fe_3_O_4_^−^TiO_2_/graphene nanocomposite as an artificial nanozymes for the detection and photodegradation of pesticides in an aqueous medium	(Boruah and Das 2020)
Soil sample	Peroxidase	Lactofen, fluroxypyr-meptyl, bensulfuron-methyl, fomesafen, and diafenthiuron	Colorimetric Detection	Nanozyme sensor arrays based on heteroatoms-doped graphene	(Zhu et al. 2020)
Water samples such as (river, tube well, and pond water)	Peroxidase	Simazine	Colorimetric detection & photocatalytic degradation	Using magnetic Fe_3_O_4_ NPs on the surface of polydopamine functionalized rGO sheets as a nanozyme	(Boruah et al. 2021)

As demonstrated in Table 4, many nonenzymatic systems for pesticide and cholinesterase (ChE) detection primarily utilize the colorimetric mode. While this mode offers the advantage of straightforward signal reading and result visualization, its sensitivity may be insufficient for the analysis of low-abundance targets. To enhance detection capabilities, more advanced techniques such as fluorescence, electrochemistry, photo electrochemistry, and others should be combined with nonenzymatic catalysis. For instance, some products resulting from nanozyme catalysis can interact with additional species that possess fluorescent properties. These interactions can either enhance or quench the luminescence of the latter through internal filtering effects, enabling the conversion of photosensitive electrons, Förster resonance energy transfer, aggregation-induced emission, intramolecular compensation, and more. This provides a foundation for target detection using the fluorescence mode, which offers higher sensitivity (M. Wang et al. 2021a, b, c).

## Air purification

### Formaldehyde

Formaldehyde (HCHO), a utilitarian chemical compound, finds frequent application in the synthesis of artificial plastics, the tanning of leather, and the preservation of tissues (El Sayed et al. 2016). Active formaldehyde can engage in reactions with olefins in the presence of specific catalysts. Alternatively, it can undergo oxidation, yielding formic acid and carbon dioxide, or it can be reduced to methanol, with both processes achievable through metal or metal oxide catalysis (Deng et al. 2020, 2014). Moreover, formaldehyde is acknowledged as a gaseous pollutant with mutagenic and carcinogenic attributes. It demonstrates strong interactions with DNA (Pontel et al. 2015), proteins, and other biomolecules, leading to noteworthy alterations in their biological functionalities (Cole et al. 2010). Based on current scientific research, when indoor formaldehyde concentration exceeds 0.1 mg/m^3^, individuals may experience an unusual odor and discomfort. These symptoms encompass eye irritation, throat discomfort or pain, nausea, throat ache, vomiting, coughing up blood, chest constriction, emphysema, asthma, and in severe cases, even sudden death (Tong et al. 2015). The importance of nanozyme in the detection and removal of formaldehyde from the air by very smart methods and these include:

#### Nano-ecological-enzyme air purification material

A newly developed specialized material, termed “nano-ecological-enzyme air purification material,” exhibits outstanding efficacy in purification, minimal wind resistance, and sterilization capabilities. This material is meticulously designed for the elimination of indoor dust, bacteria, formaldehyde, and various volatile organic pollutants, rendering it suitable for integration into air purification systems (Meng et al. 2020). The substance comprises porous polymer composites and activated carbon fiber (ACF), enriched with nano-silver and an eco-enzyme catalyst. When housed within the nanoporous carbon structure, the eco-enzymatic catalysts amalgamate into composite macromolecules containing active oxygen carriers. Comparable to the widespread occurrence of peroxidase in nature, the enzymatic catalysts within the nanopores interact with oxygen, generating highly reactive superoxide ions and active sites for oxidation–reduction (redox) reactions. The nanopores facilitate extensive interaction between the eco-enzyme catalyst and the adsorbed formaldehyde. Through a sequence of catalytic oxidation–reduction processes, the active catalyst molecule rapidly associates with the formaldehyde molecule, leading to the formation of several intermediate peroxide molecules. Ultimately, the formaldehyde undergoes conversion into water and carbon dioxide.

#### ***Detection of formaldehyde by MnO***_***2***_*** nanosheets that mimic oxidase activity.***

Due to its ability to undergo reduction and oxidation, formaldehyde can be detected using spectrophotometry, chromatography, electrochemical methods, and various other techniques (Wahed et al. 2016; Wei et al. 2018; Zhang et al. 2014). Most of these techniques, however, have limitations. They are only capable of detecting formaldehyde in air or samples with higher HCHO concentrations. Additionally, they often lack anti-interference capability and stability.

The distinctive capability of fluorescent nanoplatforms to transform chemical interactions between molecules into fluorescence signals enables the selective labeling of specific molecules or ions. This offers the advantages of high sensitivity, user-friendliness, and cost-effectiveness. Consequently, chemical and biological analytical research has recently focused on this technique (Grattieri & Minteer 2018; Katz et al. 2001). MnO_2_ nanosheets have garnered significant interest in the field of fluorescence sensing due to their favorable surface properties, broad absorption band, high redox capabilities, and excellent biocompatibility. For example, in the detection of human serum alkaline phosphatase (ALP), researchers have employed MnO_2_-fluorescent polydopamine nanoparticles. Furthermore, MnO_2_-AuNCs have displayed promise in the determination of H_2_O_2_ through fluorescence resonance energy transfer. Another intriguing attribute that has captured substantial attention is the peroxidase-like activity exhibited by MnO_2_ nanosheets or nanoflakes (Chu et al. 2020; Gu et al. 2020).

**Fig. 5:**
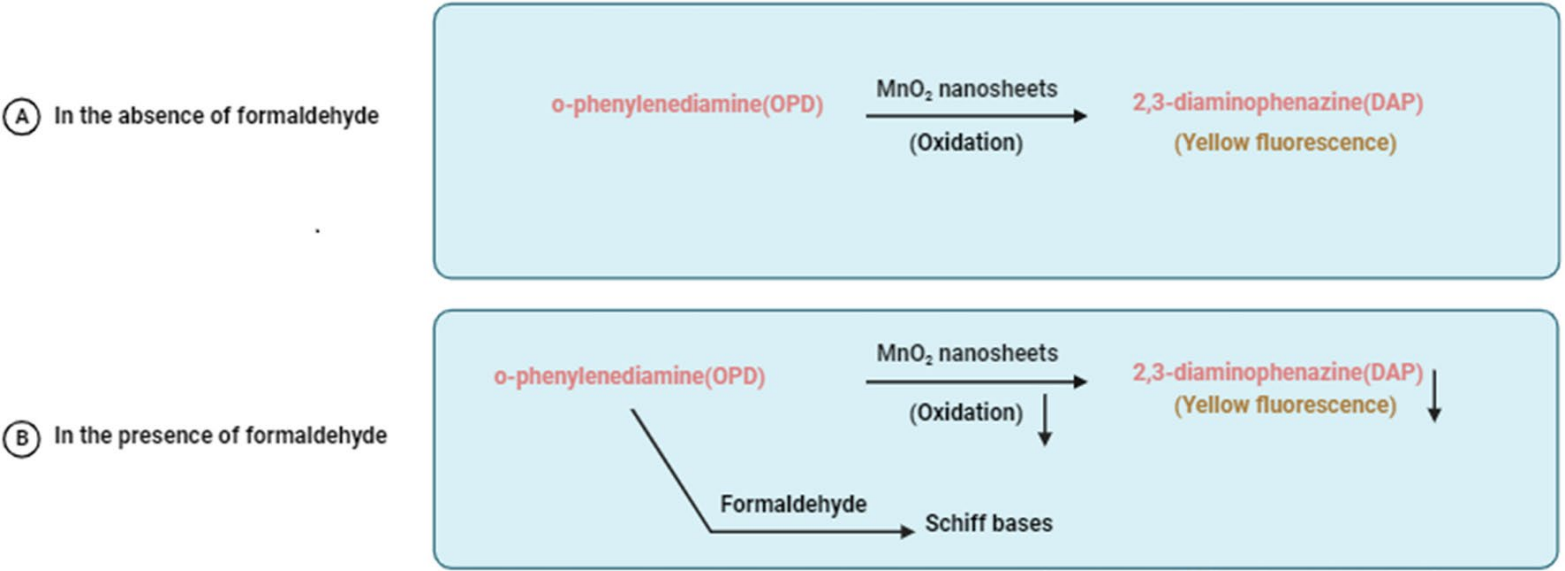
In the absence of formaldehyde, MnO_2_ nanosheets, with their oxidase-mimicking activity, expedite the oxidation of o-phenylenediamine (OPD) to produce the yellow compound 2,3-diaminophenazine (DAP), emitting a vibrant yellow color. Conversely, when formaldehyde is present, it reacts with OPD to form Schiff bases, impeding the oxidation reaction, and consequently leading to a substantial reduction in the intensity of the yellow fluorescence

In the bottom-up synthesis approach, manganese salt served as the precursor to generate MnO_2_ nanosheets, subsequently exfoliated with bovine serum albumin. The resulting nanoplatform was established by combining in situ-formed fluorescent probes of 2,3-diaminophenazine (DAP), derived from OPD, with MnO_2_ nanosheets exhibiting oxidase (OXD)-like activity. This nanoplatform demonstrates outstanding sensitivity and selectivity in detecting formaldehyde. In the presence of oxygen, MnO_2_ nanosheets facilitate the oxidation process of OPD to DAP, leading to a vibrant yellow fluorescence at a wavelength of 560 nm (Fig. 5). However, when formaldehyde is present, the interaction between OPD and formaldehyde reduces the concentration of unreacted OPD stimulated by MnO_2_ nanosheets, consequently diminishing the overall fluorescence intensity of the system (Fig. 5). The sensitive determination of formaldehyde relies on the specific Schiff base reaction between OPD and formaldehyde, which restricts the oxidation of OPD and results in a reduction in the formation of fluorescent species (Zhao et al. 2021).

### Carbon dioxide

**Fig. 6:**
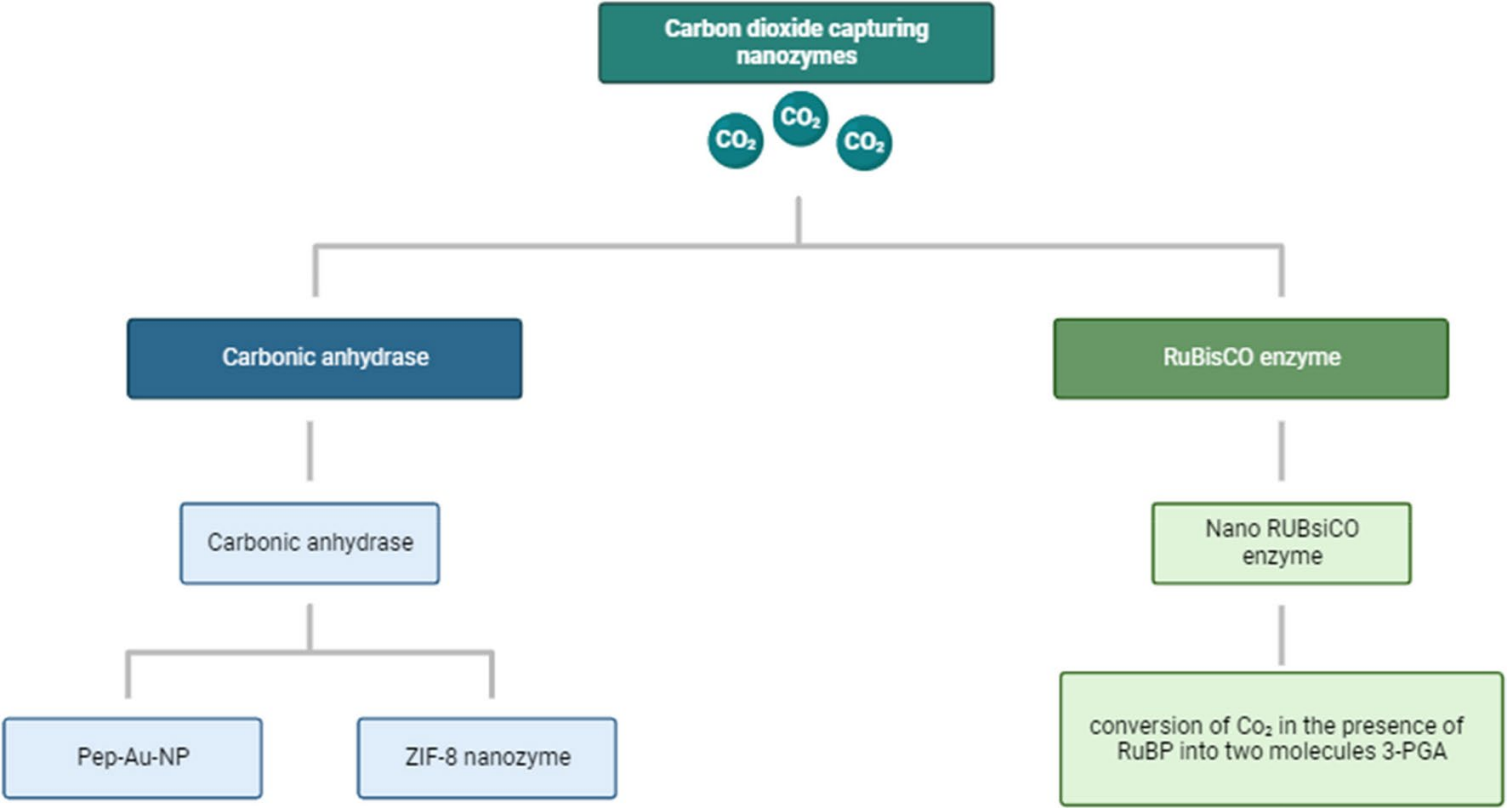
Represents Co_2_ degrading enzymes. The RuBisCO enzyme possesses a single nanozyme, known as nanoRuBisCO enzyme. This nanozyme has the capability to catalyze the conversion of CO_2_ in the presence of ribulose 1,5-biphosphate (RuBP), resulting in the production of two molecules of 3-phosphoglycerate (3-PGA). On the other hand, the carbonic anhydrase enzyme is equipped with two nanozymes: the peptide–gold nanoparticle conjugate (Pep-Au-NP) and the zeolitic imidazole framework-8 (ZIF-8). These nanozymes can catalyze the hydration of CO_2_ into hydrogen carbonate (HCO.^3−^)

The growth of industrialization and economic expansion results in an increase in planetary carbon dioxide (CO_2_) emissions. Studies indicate that heightened industrial activity correlates with elevated atmospheric CO_2_ levels. Specifically, 90% of greenhouse gases from sera, 40% from the electricity sector, 25% from natural gas processing, and widespread industrial operations in sectors like cement production, iron and steel manufacturing, and petroleum refining contribute significantly to CO_2_ emissions. If humans do not develop methods for processing or utilizing atmospheric CO_2_, the long-term rise in CO_2_ levels could lead to climate change. As an alternative to natural enzymes, nanozymes can be employed for carbon dioxide monitoring. This is achieved by harnessing their catalytic activity, which mimics that of enzymes, for CO_2_ capture (Fig. 6).

#### ***Elimination of CO***_***2***_*** by peptide–gold nanoparticle conjugate***

An effective method for producing nanozymes entails the self-assembly of thiolated ligands on the external surface of gold nanoparticles (Au-NPs). This process leads to the creation of gold clusters enclosed within a monolayer, often referred to as Au-MPCs (Pasquato et al. 2004). Broadening the domain of synthetic enzymes, peptide-gold nanoparticle conjugates (Pep-Au-NPs) have emerged as a promising path, where peptides are attached to the surface of gold nanoparticles (Mikolajczak et al. 2017; Mikolajczak & Koksch 2018; Pengo et al. 2007). We will introduce a groundbreaking advancement involving a Pep-Au-NP metalloenzyme designed to replicate the catalytic function of carbonic anhydrase (CA). The natural zinc metalloenzyme carbonic anhydrase efficiently and irreversibly catalyzes the conversion of CO_2_ into hydrogen carbonate (HCO^3−^). This remarkable activity of CA originates from its active site, which includes a zinc (Zn (II)) ion coordinated by three histidine imidazole molecules, leaving room for a water molecule to attach. This active site not only reduces the pKa of the bound water but also generates a highly nucleophilic hydroxide species, facilitated by Zn(II), which interacts with nearby bound CO_2_, resulting in the formation of HCO^−3^ (Christianson & Fierke 1996).

The development of carbonic anhydrase (CA) activity requires a peptide monolayer on a catalytically active Pep-Au-NP capable of binding to Zn (II). In this investigation, a modified version of the IHIHIQI-peptide (IHQ) was employed as the ligand for spherical Au-NPs, creating a 3-His Zn (II)-binding site between peptide strands. Notably, the peptide-gold nanoparticle conjugates (Au@IHQ-NP) exhibited significantly enhanced rates of hydrolysis for 4-nitrophenyl acetate (4-NPA) ester and CO_2_ hydration compared to their unconjugated peptide counterparts. The catalyst’s recyclability was demonstrated through five cycles of CO_2_ hydration, with Au@IHQ-NP retaining at least 94% of its original activity. The potential of IHQ-NP (a modified peptide sequence) and Au@IHQ-NP (IHQ-NP combined with Au-NPs) to mimic esterase and CA activity was investigated. In the absence of Zn (II), both IHQ-NP and Au@IHQ-NP displayed notable ester hydrolysis activity and weak catalytic capability for CO_2_ hydration. However, in the presence of Zn (II), both processes exhibited significantly improved catalytic efficiency and enhanced reaction rates. Remarkably, Zn (II)-Au@IHQ-NP demonstrated even greater rate enhancements for ester hydrolysis and CO_2_ hydration compared to the unconjugated Zn (II)-IHQ-NP variant. These findings align with prior research, underscoring the ability of Pep-Au-NPs to establish secondary coordination spheres, resulting in cooperative substrate binding, synergistic effects, and superior catalytic performance compared to unconjugated peptides (Mikolajczak & Koksch 2019).

The cumulative findings derived from this study provide compelling evidence, illustrating the potential to create Pep-Au-NPs with the ability to attract metal ions within their peptide monolayer, thus enabling metalloenzymatic activity. The results presented here introduce a design strategy based on the distinctive secondary structure of peptides, highlighting their considerable potential for future development. Through the integration of Au-NPs with catalytically active peptide sequences, a catalytic toolbox is established, holding the promise of further diversification, drawing inspiration from nature’s own examples.

#### Zeolitic imidazole framework-8 nanozyme

A bionic zeolitic imidazolate framework, known as Zeolitic Imidazole Framework-8 (ZIF-8), has been engineered as a nanozyme. Notably, the active center of human carbonic anhydrase II (hCAII), involved in the enzymatic capture of CO_2_, exhibits a geometric structure resembling the repeating unit of ZIF-8. Beyond its hCA-like activity, ZIF-8 nanoparticles also demonstrate versatile esterase and acetylcholinesterase-like activities. The active site of hCAII, Zn(His)_3_O, comprises a Zn^2+^ ion coordinated to three histidine imidazoles in a tetrahedral manner (Lipton et al. 2004). The coordination to the Lewis acid Zn^2+^ center offers two advantages. Firstly, it lowers the pKa of the H2O molecule from 14.2 to 6.8. Secondly, it facilitates the synthesis of zinc-bound hydroxide. ZIF-8, akin to the Zn(His)_3_O site of hCAII, comprises four 2-methylimidazolate (2-mIM) ligands surrounding the central Zn^2+^ ions. The tetrahedrally coordinated Zn^2+^ ions are interconnected via 2-mIM, forming a microporous framework featuring the sodalite topology (Park et al. 2006).

To assess the CA activity and examine the similarities between p-nitrophenyl acetate (pNPA) hydrolysis and CO_2_ hydration reactions, pNPA was initially employed as the colorimetric substrate (Casey et al. 2014; Rufo et al. 2014; Zastrow et al. 2012). The concentrations of ZIF-8 and the substrate had a significant impact on the rate at which pNPA was hydrolyzed. Increasing either the concentration of ZIF-8 or pNPA notably accelerated the reaction rate (J. Chen et al. 2019a, b).

Hydrogen ions are produced during the CO_2_ hydration reaction, which raises the solution’s acidity. As a result, the pH change in the buffer solution can be used to determine the hydration of CO_2_.

$${{\text{CO}}}_{2}+{{\text{H}}}_{2}{\text{O}}\to {{\text{HCO}}}_{3-}+{{\text{H}}}^{+}$$

Due to the neutralization of the alkaline solution, which specifically promotes CO_2_ hydration, a slight decrease in pH from 8.3 to 7.6 was observed in the pH decay of a control experiment. The esterase experiment demonstrates that ZIF-8 can effectively accelerate CO_2_ hydration, causing the pH value to rapidly decrease to 6.9 upon the application of the nanozyme. Furthermore, ZIF-8 nanoparticles exhibit exceptional durability for reuse as a potent nanozyme. The ZIF-8 nanozyme holds the potential for further enhancement to facilitate effective catalysis in a range of processes. This study serves as an example of a biomimetic approach to designing and synthesizing efficient metal–organic frameworks (MOFs) nanozymes at the molecular level (J. Chen et al. 2019a, b).

#### Nano RuBisCO enzyme

In the Calvin cycle, a key enzyme in the process is ribulose bisphosphate carboxylase/oxygenase (RuBisCO). This enzyme plays a vital role in the conversion of CO_2_ and ribulose 1,5-bisphosphate (RuBP), which initiates the synthesis of essential molecules like glucose (Feller et al. 2008). The carbon fixation process is vital for plants as it allows them to convert CO2 into glucose, and RuBisCO serves as a catalyst in the carboxylation of RuBP. When CO2 is the substrate, RuBisCO’s carboxylase activity results in the formation of a highly unstable 6-carbon phosphorylated intermediate known as 3-keto-2-carboxyarabinitol-1,5-bisphosphate. The hydrolysis of this intermediate produces two molecules of 3-phosphoglycerate (3-PGA), which act as precursor molecules for the synthesis of larger compounds like glucose (Erb & Zarzycki 2018; Phillips & Milo 2009; Wilkes & Pearson 2019). The enzymatic function of RuBisCO is dependent on the presence of magnesium ions (Mg2 +). Within the enzyme’s active site, CO2 can be activated, leading to the acquisition of a carbamate group by a lysine residue. This process is facilitated by the precise positioning of Mg2 + ions. The concentration of Mg2 + ions and pH variations play crucial roles in regulating RuBisCO activity. These factors are notably influenced by an increase in concentration within the chloroplast stroma when exposed to light (Andersson 2008; Berry et al. 2016, 2013).

Within the realm of this chemistry study, the RuBisCO enzyme assumes a pivotal role in the efficient uptake of CO_2_ molecules within plant cells. It facilitates the fusion of CO_2_ with five-carbon sugar molecules, yielding a novel six-carbon sugar compound vital for the metabolic processes of plant cells. To enhance the enduring stability and performance of the RuBisCO enzyme during the CO_2_-to-sugar conversion, a nanostructured version of the enzyme has been devised for in vitro applications. This nanostructured enzyme, denoted as nanoRuBisCO, has been engineered using the ANADOLUCA (AmiNoAcid Decorated and Light Underpinning Conjugation Approach) technique. It boasts adaptability across a broad spectrum of pH and temperature conditions (Say et al. 2011).

The ANADOLUCA methodology has facilitated the creation of nanoRuBisCO enzymes that showcase enhanced stability, efficiency, and reusability when juxtaposed with the unbound RuBisCO enzyme. Noteworthy is the pioneering aspect of this research, being the first to introduce the nanoRuBisCO enzyme in scientific literature. All experimental data were rigorously compared against those acquired using the unbound RuBisCO enzyme as the benchmark. The outcomes unmistakably affirm the nanoRuBisCO enzyme’s ability to catalytically transmute CO_2_ into 3-PGA, thereby substantiating its functional prowess. Furthermore, the enzyme activity was gauged through spectrophotometric measurements, an advancement that bestows an additional benefit upon the devised system. As per the findings, the unbound RuBisCO enzyme exhibited an enzyme activity of 8.62 U/mg of substrate, while the nanoRuBisCO enzyme displayed an enzyme activity of 6.50 U/mg of substrate (Biçen Ünlüer et al. 2021). According to various sampling dates, RuBisCO enzyme activity in tomato plants was reported as 4–9 U/mg substrate in the literature (Mitra et al. 2019). In an independent investigation, various experimental conditions were explored to study the factors influencing the activity of the RuBisCO enzyme. The results of this study demonstrated that the enzyme activity of RuBisCO varied within the range of 6 to 10 U/mg of substrate (Zhang et al. 2020a, b).

Biçen Ünlüer et al. marks a pioneering achievement in the production of the RuBisCO nanozyme, distinguishing this study from its predecessors and setting it apart from the conventional RuBisCO enzymes discussed in the source literature. Notably, this newly created nano enzyme displayed exceptional recyclability, enduring up to 23 cycles without structural deterioration. The experimental conditions governing enzymatic activity were streamlined, rendering them accessible for a diverse array of applications when RuBisCO enzymes are synthesized in nanostructures. This nanozyme exhibits significant potential for catalyzing CO_2_ conversion into various beneficial chemical processes, as well as for research pertaining to carbon capture and carbon fixation, mirroring the principles of photosynthesis.

### Reactive oxygen species from cigarette smoke

Tobacco use is acknowledged as a risk factor for most of the top eight global causes of mortality (Wipfli & Samet 2016). Reactive oxygen species (ROS), including harmful compounds like hydrogen peroxide and oxygen-containing radicals, are generated within smoked cigarettes. Current cigarette filter technology does not efficiently eliminate these ROS (Korschelt et al. 2017). ROS seriously harms the immune system and lung tissue (Onizawa et al. 2009; Stämpfli & Anderson 2009), which results in several illnesses (including lung cancer and chronic obstructive pulmonary disease).

Antioxidants, including catalytic antioxidants, have previously been integrated into cigarette filters. For example, to mitigate the presence of reactive oxygen species (ROS) in cigarette smoke, antioxidants derived from plants such as lycopene, pycnogenol, and grape seed extract have been introduced into smoking filters (Shen et al. 2010; Zhang et al. 2002). However, due to their poor thermal stability and limited antioxidant capacity, these substances exhibit weak scavenging abilities. Consequently, catalytic antioxidants have recently gained attention as potential substitutes for conventional antioxidants.

To counteract elevated ROS levels, intracellular enzymes, such as glutathione peroxidase, catalase, and superoxide dismutase (SOD), have been developed. Nanozymes have displayed enhanced stability and resilience under harsh conditions. Consequently, investigations into highly active and thermally stable antioxidant enzyme-mimicking nanozymes offer promising avenues for mitigating ROS in cigarette smoke and reducing associated harm.

#### Copper tannic acid coordination nanosheet

**Fig. 7:**
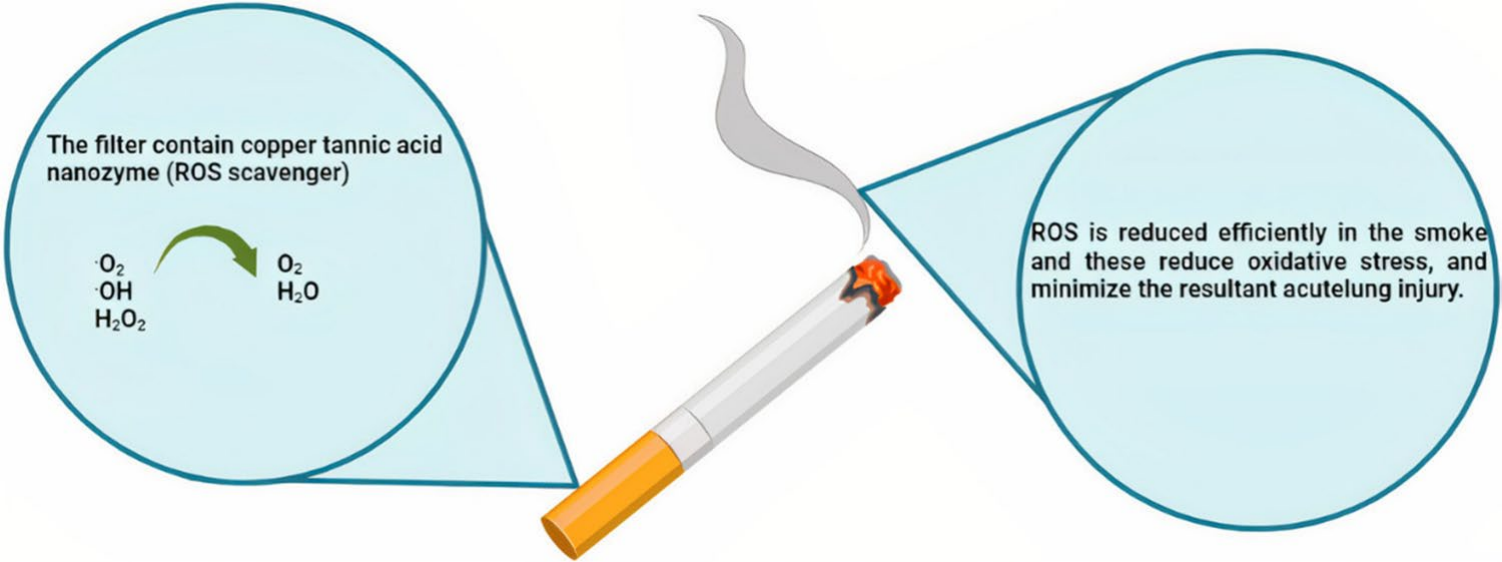
Copper tannic acid coordination (CuTA) nanozyme exhibits intrinsic catalase-like activity, hydroxyl radical elimination capacity, superoxide dismutase-like activity, and can capture ROS in the cigarette smoke

This study utilized copper coordination with tannic acid to fabricate an antioxidant nanozyme capable of scavenging ROS, inspired by antioxidant enzymes. The newly created coordination nanosheets composed of copper and tannic acid (CuTA) nanozyme exhibited catalase and SOD-like activities, along with the ability to eliminate hydroxyl radicals (•OH). Thanks to its distinctive coordination structure, the CuTA nanozyme possessed a high degree of heat stability. It demonstrated remarkable antioxidant properties, making it highly efficient in ROS scavenging. Leveraging these attributes, the CuTA nanozyme was applied to modify cigarette filters. Coating the commercial cigarette filter with the CuTA nanozyme provided protection to mice exposed to cigarette smoke, reducing pulmonary inflammation and acute lung injury. The CuTA nanozyme exhibited effective ROS scavenging capabilities within cigarette smoke (Fig. 7). The development of the CuTA nanozyme introduces a novel approach to enhance cigarette filters, mitigating the adverse effects of cigarette smoke. Moreover, it offers a versatile ROS scavenger with diverse antioxidant capabilities (Lin et al. 2020b, a).

## Role of nanozymes in pathogens

The World Health Organization estimates that infectious diseases account for over 25% of global fatalities, with the majority attributed to bacteria and viruses. These diseases can be transmitted through contaminated food and water. Thus, the initial measure in managing infectious diseases involves the detection of pathogenic microorganisms (Zhang et al. 2018a, b). The utilization of nanozymes in biosensing has undergone recent expansion, propelled by the development and synthesis of various nanozyme-based systems designed as probes for the detection of bacteria and viruses (T. Zhang et al. 2019a, b). To illustrate, for the identification of the rubella virus, Au@Pt may be employed within a nanozyme-linked immunosorbent assay (NLISA). Furthermore, a naked-eye immunoassay for the colorimetric detection of *Staphylococcus aureus* has been devised using peroxidase-like CuMOF NPs (Wang et al. 2017).

Foodborne pathogens are typically found in contaminated or undercooked food, posing a significant health threat that has even resulted in fatalities on occasion. Commonly identified pathogens like *Escherichia coli* O157:H7, *Salmonella enteritidis*, *Listeria monocytogenes*, and *Vibrio parahaemolyticus* can be encountered in food items. Detecting these pathogens through straightforward means is essential. Additionally, viruses exhibit remarkable robustness and possess a high degree of transmissibility. Moreover, their capacity for rapid mutation and genetic recombination significantly heightens the risk of a pandemic, especially in our intricately connected global society. This underscores the urgent need for rapid and highly sensitive detection techniques designed to target pathogenic agents.

### *Escherichia coli*

The effective deployment of control measures hinges on the utilization of adept, swift, and exceptionally sensitive detection techniques precisely engineered for targeting pathogenic bacteria (Scallan et al. 2011). We devised a detection strategy employing nanozymes in a cascade reaction. In an acidic solution, MnO_2_ nanosheets displayed catalytic activity, transforming the colorless TMB substrate into TMB^2+^ ions. TMB^2+^, serving as an oxidant, swiftly eroded Au NRs, leading to discernible color alterations. In our sensing technique, β-galactosidase derived from *E. coli* instigated this cascade reaction. β-galactosidase facilitated the hydrolysis of the p-aminophenol β-D-galactopyranoside (PAPG) substrate, yielding a reducing agent named p-aminophenol (PAP). PAP was identified as a MnO_2_ nanosheet reducer, hindering their oxidase activity and subsequently diminishing TMB^2+^ production. Consequently, the etching process of Au NRs was hindered. The various colors resulting from Au NR etching enabled a semi-quantitative visual analysis of β-galactosidase in *Escherichia coli*. The synergistic application of enzyme-nanozyme cascade reactions and the unique optical characteristics of Au NRs significantly amplified the detection sensitivity for β-galactosidase in *E. coli*. Furthermore, this approach furnished a high-resolution and user-friendly visual means for determining *E. coli* concentration via direct naked-eye observation (Zhou et al. 2020).

In a separate investigation, *Escherichia coli* (*E. coli*) was detected using a fluorescent-based immunosensor technique. Within this immunosensor, magnetic beads and FITC were appropriately labeled with antibodies that were conjugated to the target *E. coli*, forming a sandwich-like structure. When subjected to a blue laser light (405 nm), this sandwich structure generated a fluorescent signal in the presence of the target pathogen. An iPhone 5S, coupled with ImageJ software, was effectively employed to convert the fluorescence image into fluorescence intensity, enabling the quantification of *E. coli*. The limit of detection (LOD) for *E. coli* in yogurt samples was 1 CFU mL^−1^, while in egg samples, it was 10 CFU mL^−1^ (Zeinhom et al. 2018a, b). Zheng and colleagues introduced an approach that integrates a microfluidic chip, immunoassay, and smartphone for *Escherichia coli* (*E. coli*) detection. In this methodology, the HSL-based imaging application was deployed to quantify the *E. coli* concentration in chicken samples, with a limit of detection (LOD) set at 50 CFU mL^−1^ (Zheng et al. 2019).

### *Pseudomonas aeruginosa*

*Pseudomonas aeruginosa* (PA) is an opportunistic Gram-negative pathogen with substantial clinical importance, given its ability to provoke a diverse array of severe nosocomial bloodstream infections (Leung et al. 2017). As per the guidelines established by the World Health Organization (WHO), *Pseudomonas aeruginosa* (PA) is acknowledged as an indicator organism for assessing the quality of drinking water. Moreover, owing to its widespread prevalence in the environment, PA stands as one of the most infamous pathogens accountable for water contamination across diverse regions globally, encompassing Asia, Europe, and the USA (Mena & Gerba 2009). Gold nanoparticles (GNPs) possess distinctive surface plasmon resonance and catalytic qualities, rendering them indispensable in the realm of colorimetric detection. Within this framework, GNPs function as catalysts, instigating the oxidation of TMB, which results in the formation of diamine. Subsequently, diamine undergoes conversion into diimine, an electroactive product of significant interest. To harness the electroactive attributes of TMB for electrochemical sensing, scientists have devised an aptamer-nanozyme-based assay employing readily available disposable screen-printed electrodes. This integration enables the acquisition of an electrochemical readout that enables the highly sensitive detection of *Pseudomonas aeruginosa* (PA). The amalgamation of the nanozyme assay with an electrochemical platform brings forth substantial improvements in diagnostic sensitivity. The aptamer-nanozyme-based electrochemical approach offers remarkable characteristics, including rapidity (within a mere 10 min), cost-effectiveness, and outstanding sensitivity (capable of detecting concentrations as low as approximately ~ 60 CFUs/mL in water). Furthermore, this platform exhibits considerable potential as a versatile detection system for a wide array of molecular and cellular analytes, thus showcasing its versatility for broader applications.

### Salmonella

The World Health Organization (WHO) has provided an estimate indicating that roughly 10% of the global population suffers from foodborne illnesses each year, resulting in the forfeiture of 33 million healthy life years annually (Drinković et al. 2004). Salmonella is one of the four primary worldwide etiological factors contributing to the incidence of diarrheal diseases (Hu et al. 2020; Jin et al. 2020). Zeinhom et al. devised a sandwich immunosensor, incorporating magnetic beads and inorganic nanoflowers, for the swift detection of *S. enteritidis*. In this system, the synthesized inorganic nanoflowers demonstrated peroxidase-like activity, leading to the generation of a blue color upon the addition of TMB. RGB color values from the captured image were subsequently extracted using ImageJ software. This newly created assay exhibited the capability to detect *S. enteritidis* in both milk and cheese, achieving a limit of detection (LOD) of 1 CFU mL^−1^ and 1 CFU/g, respectively (Zeinhom et al. 2018a, b). Furthermore, the inorganic nanoflowers demonstrated superior sensitivity compared to the Pd@Pt nanozyme in the detection of *S. enteritidis* (Cheng et al. 2017). Glucose oxidase (GOx) represents a frequently employed enzyme in biosensors, employed for the conversion of glucose into H_2_O_2_ and gluconic acid (Yang et al. 2013). Guo et al. have applied this principle to detect *S. typhimurium*. In their procedure, they synthesized immuno GOx-nanoclusters (GNCs) and affixed them to *S. typhimurium*, resulting in the formation of nanocluster bacteria (GNCBs). These GNCBs possessed the ability to catalyze glycose, inducing color development in the strip. An image of the strip was captured and processed using a custom-developed application. The hue, saturation, and lightness (HSL) color space platform were introduced to quantify *S. typhimurium* and establish a limit of detection (LOD) of 1.6 × 10 CFU mL^−1^ (Guo et al. 2019).

### Nov-LP

Norovirus (NoV) comprises a genetically varied assembly of small, icosahedral, non-enveloped viruses, featuring a genome of positive-sense, single-stranded RNA (ssRNA) (Parashar 2013). NoV exhibits a considerable degree of contagion, primarily ascribed to its remarkably low infective dose. The estimated median infective dose (ID50) falls within a range spanning from 18 to 1015 genome copies (N. Wang et al. 2021a, b, c). Currently, the most dependable and sensitive method for detecting and quantifying NoV is reverse-transcription–quantitative-polymerase-chain-reaction (RT-qPCR). Detecting NoV in food presents significant challenges, necessitating a specific virus concentration for the matrix and the removal of inhibitory substances for detection assays. Consequently, the application of RT-qPCR encounters hurdles when it comes to swift, on-site, or point-of-care diagnostic applications. In addressing this concern, Weerathunge and colleagues propose an innovative colorimetric nanozyme aptasensor strategy capable of facilitating rapid detection (within 10 min) of infectious murine norovirus (MNV) with high sensitivity. This novel method achieves a calculated limit of detection (LOD) of 3 viruses per assay (equivalent to 30 viruses/mL) and an experimentally validated LOD of 20 viruses per assay (equivalent to 200 viruses/mL) (Weerathunge et al. 2019). The introduction of this innovative assay format carries substantial implications as it allows for swift and highly sensitive detection of norovirus in a matter of minutes. Furthermore, it is user-friendly and eliminates the requirement for specialized laboratory facilities. The creation of this colorimetric nanozyme aptasensor strategy marks a noteworthy progression in the field of norovirus detection.

### RV

The rubella virus (RV), a human pathogen, is accountable for the emergence of German measles, an airborne childhood ailment recognized for its significant contagiousness and worldwide prevalence. In cases where rubella infection takes place during pregnancy, it results in the onset of congenital rubella syndrome, characterized by the established trio of cataracts, cardiac irregularities, and sensorineural deafness (Bouthry et al. 2014; Sharma & Kakkar 2016). Given the critical nature of rubella virus detection, the utilization of exceedingly sensitive and efficient detection techniques is imperative. Among traditional methodologies, employing serological testing for rubella immunoglobulin (Ig) M stands as a standardized approach for confirming acute infections (Helfand et al. 2001, 2007). Peroxidases, including horseradish peroxidase (HRP), find extensive use in enzyme-linked immunosorbent assays (ELISAs) to identify and measure antigens, antibodies, viruses, or cells. Nonetheless, HRP’s inherent instability can potentially lead to a notable incidence of false-negative results (Raynal et al. 2014). Drawing inspiration from mesoporous silica-coated nanocrystals, which effectively retain the functional attributes of their core while offering advantageous surface functionalization capabilities, Li’s research team has introduced a new nanozyme designated as Au-core@Pt-shell@mesoporous silica (Au@Pt@SiO2) for use in immunoassays. The synthesized Au@Pt@SiO2 nanozyme displays catalytic prowess in chromogenic reactions within immunoassays, thus potentially serving as a viable replacement for natural enzymes in conventional enzyme-linked immunosorbent assays (ELISAs). Moreover, a novel conjugate employing the antigen-labeled Au@Pt@SiO2 nanozyme has been devised as a nanoprobe for virus serodiagnoses. This antigen-labeled Au@Pt@SiO2 nanozyme exhibits highly sensitive peroxidase-like activity, along with impressive catalytic stability and robustness, rendering it well suited for applications in biochemical assays and clinical diagnostics (A. Li et al. 2019a, b, c). The research carried out by Li and fellow researchers, centering on the creation of the Au-core@Pt-shell@mesoporous silica nanozyme and its application as a nanoprobe for virus serodiagnosis, marks a substantial progression in the realm of rubella virus detection. The heightened stability and catalytic characteristics exhibited by this nanozyme hold considerable promise for augmenting biochemical assays and clinical diagnoses. Consequently, this contributes to advancements in the precision and dependability of rubella virus detection and diagnostic procedures.

Additionally, our focus extends to the identification of pathogenic microorganisms through the utilization of diverse nanozymes, as demonstrated in Table 5.

**Table 5:** Detection of several pathogens by different nanozymes

Nanozyme	Enzyme-like activity	Pathogen	Method	References
Au@Co-Fe NPs	Peroxidase	Bacteria (+ ,-)	Colorimetric assay method	(Mirhosseini et al. 2020)
gold nanorods	Oxidase	*Escherichia coli*	Multicolor colorimetric platform	(Zhou et al. 2020)
His-Fe_3_O_4_@Cu	Peroxidase	*Salmonella Typhimurium*	Sensitive colorimetric detection	(Wang et al. 2022a)
Pd@Pt	Peroxidase	*Staphylococcus aureus*	Colorimetric N-ELISA	(Wang et al. 2022b)
Au@Pt	Peroxidase	Streptomycin	Lateral-flow immunoassay	(Wei et al. 2020)
Silver-gold/silver chloride	Oxidase & peroxidase	Urinary spermine	Colorimetric and fluorescence assays	(Kuo et al. 2018)
Graphene Quantum Dots	Peroxidase	*Y. enterocolitica*, *Y. pestis*, and *Y. pseudotuberculosis*	Serological tests, enzyme link immunosorbent assay (ELISA), and polymerase chain reaction (PCR)-based tests	(Savas and Altintas 2019)
Mesoporous carbon-dispersed Pd nanoparticles	Peroxidase	H_2_O_2_	Several methodologies, including titrimetry, chromatography, electroanalysis, colorimetry, and fluorometry	(Zhang et al. 2018a, b)
Histidine-containing carbon nanodots	Oxidase	*Escherichia coli*	Colorimetric assay method	(Loukanov et al. 2022)
Compact Cu-anchored photothermal polydopamine	peroxidase	*Aspergillus flavus*	Colorimetric and photothermal lateral flow immunoassay	(Liang et al. 2022)
oleic acid (OA) nanoemulsion & manganese dioxide (MnO_2_	Oxidase	Bacteria	Colorimetric and fluorescence assays	(Liu et al. 2021c)
Fe-doped polydopamine (Fe@PDA)	Peroxidase	Listeria monocytogenes	Fluorescence-colorimetric dual-mode platform	(Shen et al. 2022)
Fe_3_O_4_@PDA@Pt nanocomposite	Peroxidase	*Escherichia coli*	Lateral flow immunoassay	(Dou et al. 2022)
PtCu nanoalloys (NAs)	Oxidase	Alpha-synuclein (alpha syn)	fluorescence assay	(Liu et al. 2021a)
Co–Fe@hemin	Peroxidase	SARS-CoV-2	ELISA method and nanozyme colorimetric strips	(Liu et al. 2021d)

## Role of nanozymes in antibiotics

Nanozymes are nanomaterials that exhibit enzymatic activity, resembling natural enzymes. They possess distinct advantages and characteristics, including but not limited to low cost and remarkable stability (Liao et al. 2022), Moreover, they outperform traditional methods by considerably reducing reaction times. Owing to their diminutive size, nanozymes exhibit enhanced specialization and capacity (Huang et al. 2022b, a). Nanozymes play a pivotal role in the detection and identification of antibiotic residues, which constitute one of the most perilous environmental pollutants. Their peril arises from their extended half-life relative to natural pollutants, as well as their ability to traverse the food chain and ultimately affect human health (He et al. 2020b, a). These compounds give rise to a multitude of issues, encompassing renal toxicity, intestinal infections, and allergic reactions. Furthermore, they exhibit resistance to several antibiotics, rendering the latter ineffective. In the year 2004, approximately 700,000 individuals succumbed to antibiotic resistance, and it is projected that this number may surge to 10 million by the year 2050 (Gao et al. 2021). Hence, nanozymes have become a dependable choice for antibiotic residue detection owing to their remarkable efficiency. Furthermore, certain categories of nanozymes can be modified to acquire infection control attributes, effectively transforming them into antibacterial agents. These modifications leverage their catalytic activity, enabling them to exhibit heightened effectiveness and specialization against microbial threats (D. Wang et al. 2021a, b, c). The detection of antimicrobials using nanozymes holds significant importance across numerous domains (X. Zhang et al. 2019a, b), notably in the realm of food safety, facilitated through the utilization of biosensors. This approach encompasses a diverse array of nanozymes, effectively addressing challenges inherent in conventional techniques like liquid chromatography (HPLC) and liquid chromatography-mass spectrometry (HPLC–MS) (Gaudin 2017). This technology contributes to the advancement and establishment of biosensor systems, capitalizing on its inherent advantages, including heightened efficiency and reduced time consumption, rendering it applicable across various other disciplines (Wang & Gunasekaran 2020).

Our focus will be on the detection of antibiotic residues using various nanozymes, as indicated in Table 6, as well as on modified nanozymes designed to exhibit antimicrobial properties, detailed in Table 7. These modifications introduce essential characteristics into nanozymes, enhancing their specialization in the identification and removal of antimicrobial agents. Each nanozyme undergoes a specific modification process tailored to its function and its compatibility with the bacterial antibiotics it aims to target.

**Table 6:** Detection of several antibiotic residues by different nanozymes

Antibiotic	Nanozyme	Enzyme-like activity	Method	Mechanism	References
Kanamycin	(Au NPs) (Pt NPs) (WS2 NPs)	Peroxidase	Colorimetric sensing (aptasensors)	Aptamer is used to bind with nanozyme after being processed with a metal–organic framework and added to the antibiotic and then form a compound between the aptamer and the antibiotic due to the great bonding force between them and its detection	(Li et al. 2022a, b, c)
Chloramphenicol (CAP)	Co_3_O_4_	Peroxidase	Electrochemiluminescence (ECL)	Covalent organic framework was constructed and combined with aggregation induced emission Thus nanozyme can be amplified and enables better selectivity to CAP	(Li et al. 2021)
Enrofloxacin (ENR)	Co(OH)_2_	Peroxidase	Chemiluminescence immunoassay	Resin from carboxyl is used to provide more surface area to carry ENR according to competitive immune assay theory, which increases the speed and sensitivity of detection	(Pei et al. 2021)
MicroRNA	Au@CD NPs	Peroxidase	Electrochemiluminescence (ECL) Photoelectrochemical (PEC)	TiO2-CFPE and CeO_2_-CFPE are used as an electrical agent to increase nanozyme’s cleavage time and adjust its concentrations, giving it greater sensitivity that enables it to detect	(He et al. 2020a)
Sulfadiazine	Au@Pt@SiO_2_	Peroxidase	Biomimetic Immunoassay	Nanozyme acts as a marker because it has high catalytic activity that helps in the process of electrochemical catalysis and catalytic hydrogenation, which makes it more efficient for detection sulfadiazine residue	(He et al. 2020b)
Sulfamethazine (SMZ)	PtNi	Peroxidase	Photoelectrochemical (PEC)	Bimetallic PtNi nanozyme is built and used as a signal amplifier for easy SMZ detection	(Song et al. 2022)

**Table 7:** Modified nanozymes that act as antimicrobial

Microbe	Nanozyme	Enzyme-like activity	Modification	References
Methicillin-resistant *Staphylococcus aureus* (MRSA)	CuCo_2_S_4_	Peroxidase	Nanozyme was developed by dextran to resist bacteria and also mimics the enzyme at neutral pHs that cause it to convert H_2_O_2_ into OH to fight bacteria and inhibit resistant bacteria	(Li et al. 2020)
Ampicillin-resistant *Escherichia coli* and methicillin-resistant *Staphylococcus aureus*	UNMS NCs	Peroxidase	Nanozyme is positively charged to increase its enzymatic properties making it more able to pick up and restrict bacteria	(Liao et al. 2022)
*E. coli*	Fe_3_O_4_@MoS_2_-Ag	Peroxidase	This nanozyme was developed to have a rough surface filled with burrs which helps it capture bacteria further and then release Ag that attacks the bacteria membrane	(Wei et al. 2021)
SARS-CoV2	Ag-TiO_2_ SAN	Peroxidase	The combination of TiO2 and Ag gave the compound greater specialization, higher ability to absorb and link to the virus and the ability to stop it	(Wang et al. 2021a, b, c)
Methicillin-resistant *Staphylococcus aureus* and hyperspectral bacteria β-*Escherichia coli*	CuFeS_2_	Peroxidase	Nanozyme, after photothermal treatment, changes the permeability of the membrane of bacteria cells and disrupts the antioxidant balance because it consumes the glutathione (GSH) in the cells to produce ROS in an appropriate way that kills bacteria	(Liu et al. 2022)

## Future prospective

1. The development of nanozymes with novel catalytic capabilities, such as hydrolase and synthetase, should be the focus of future research given the diversity of natural enzymes.
2. Natural enzymes typically function in groups called enzyme clusters. Many efforts will be made to integrate the features and capabilities of nanozymes with natural enzyme assemblies to replicate the intricate natural enzyme systems.
3. The practical applications of nanozymes must also be expanded by synergistically combining them with other functional nanoparticles that have unique nanoscale features, such as magnetics, optical, electrics, and mechanics.
4. Seeking environmentally safe nanozymes for application is crucial. The toxicity of nanozymes itself should be taken into consideration when used in environmental analysis. It is well known that due to possible environmental and health risks, the toxicity of nanomaterials is receiving more and more attention from the academic community and the public.
5. Exploring why, although quite different in terms of content, conformation, and shape from genuine enzymes, nanoparticles might mimic them is necessary. More research is required on the intricate catalytic mechanisms and theories underlying these intriguing events.

## Conclusion

In this review, we have discussed the recent applications based on nanozyme technology for sensing and removing pollutants from the environment. Nanozymes are a prospective replacement for natural enzymes due to their benefits of great stability, customized features, simple mass production, and low cost. Although a lot of development has already been achieved in the field of nanozymes, there are still many problems, barriers, and challenges to overcome.

## Challenges

1. Enhancing the selectivity of nanozymes poses a formidable challenge for future endeavors, given their limited ability to catalyze specific substrates akin to natural enzymes.
2. Despite the fabrication of several successful nanozyme-based sensing systems for detecting specific pollutants, the range and diversity of targets that can be identified by nanozymes remain considerably constrained.
3. Within current sensing systems, the majority of nanozymes lack recyclability and environmental compatibility. Consequently, there is an inevitable trend in the scientific community to explore environmentally friendly and recyclable nanozymes for the purpose of pollutant detection.
4. Despite evading many inhibitory factors present in natural enzymes, the catalytic activities of most nanozymes still fall considerably short when compared to their naturally occurring counterparts.
5. Despite the high efficacy of pollutant treatment, the industrial cost associated with this approach may exceed that of traditional pollution treatment methods.
6. While nanozymes have demonstrated excellent performance in small-scale experiments, their utilization in environmental engineering remains limited. This is primarily attributed to the requirement for highly precise technological advancements and extended operational lifespans in catalytic nanozyme devices. However, should these drawbacks be addressed, the utilization of nanozymes in the pollutant treatment industry could offer significant advantages.
